# Evaluating the Performance of a Non-Bonded Cu^2+^ Model Including Jahn−Teller Effect into the Binding of Tyrosinase Inhibitors

**DOI:** 10.3390/ijms21134783

**Published:** 2020-07-06

**Authors:** Lucas Sousa Martins, Jerônimo Lameira, Hendrik G. Kruger, Cláudio Nahum Alves, José Rogério A. Silva

**Affiliations:** 1 Laboratório de Planejamento e Desenvolvimento de Fármacos, Instituto de Ciências Exatas e Naturais, Universidade Federal do Pará, Belém, Pará 66075-110, Brazil; lucas-desouza-30@hotmail.com (L.S.M.); lameira@ufpa.br (J.L.); nahum@ufpa.br (C.N.A.); 2 Catalysis and Peptide Research Unit, University of KwaZulu-Natal, Durban 4000, South Africa; Kruger@ukzn.ac.za

**Keywords:** tyrosinase, inhibition, kojic acid, Jahn-Teller effect, binding free energy, computational chemistry

## Abstract

Tyrosinase (TYR) is a metalloenzyme classified as a type-3 copper protein, which is involved in the synthesis of melanin through a catalytic process beginning with the conversion of the amino acid l-Tyrosine (l-Tyr) to l-3,4-dihydroxyphenylalanine (l-DOPA). It plays an important role in the mechanism of melanogenesis in various organisms including mammals, plants, and fungi. Herein, we used a combination of computational molecular modeling techniques including molecular dynamic (MD) simulations and the linear interaction energy (LIE) model to evaluate the binding free energy of a set of analogs of kojic acid (KA) in complex with TYR. For the MD simulations, we used a dummy model including the description of the Jahn–Teller effect for Cu^2+^ ions in the active site of this enzyme. Our results show that the LIE model predicts the TYR binding affinities of the inhibitor in close agreement to experimental results. Overall, we demonstrate that the classical model provides a suitable description of the main interactions between analogs of KA and Cu^2+^ ions in the active site of TYR.

## 1. Introduction

Melanin is present in many organisms where it plays several key roles including photoprotection, thermoregulation, and wound healing. In humans, the melanin pigment is also responsible for the color of skin, eyes, and hair [1,2,3]. The abnormal loss of melanin and depigmentation can be a serious facial aesthetic and dermatological problem among humans [4]. On the other hand, increasing melanin synthesis and accumulation of these pigments occur in many types of skin disorders, including neurodegeneration associated with Parkinson’s disease and skin cancer [5,6,7]. Tyrosinase (TYR) is one of the key enzymes in mammalian melanin synthesis [8] and its function in melanin biosynthesis is well documented [9]. A recent article has reviewed the importance of inhibitors of TYR, which are utilized in cosmetic and medicinal industries as depigmentation agents and also in food and agriculture industries as antibrowning compounds [10]. Therefore, inhibitors of TYR are a class of important clinical drugs.

**Figure 1:**
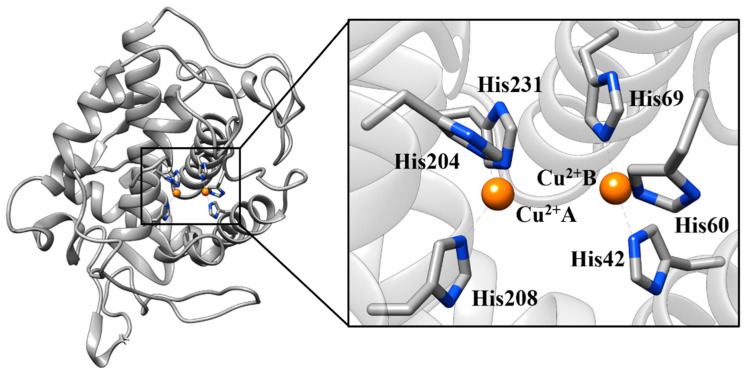
The catalytic site of tyrosinase (TYR) where Cu^2+^ ions interact with key histidine residues (Protein Data Bank (PDB) code 5I38).

**Figure 2:**
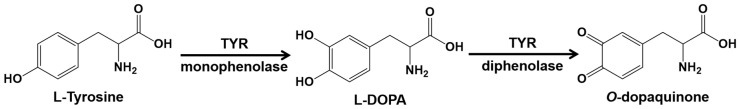
Melanogenesis stages catalyzed by the tyrosinase (TYR) enzyme.

TYR is a metalloenzyme found in animal tissues, mushrooms, and plants [11,12,13]. There are two copper ions (Cu^2+^) in the active site of tyrosinase. Each Cu^2+^ coordinates with three histidine residues (Figure 1) [14,15]. In its catalytic cycle (Figure 2), the l-Tyrosine (l-Tyr) is hydroxylated to l-3,4-dihydroxyphenylalanine (l-DOPA), a phase called monophenolase, and then l-DOPA is oxidized to o-dopaquinone, a phase called diphenolase [1,16]. Melanin is then rapidly formed by the spontaneous polymerization of o-dopaquinones [15,17].

Examples of TYR inhibitors are kojic acid (KA) [18], tropolone (TRO) [19], coumarins [20], vanillic acid, vanillin, and vanillic alcohol [21]. Particularly, KA is used as a positive standard in experimental measurements [22,23]. However, it has been reported that kojic acid has side effects such as a high sensitization potential, causing irritant contact dermatitis and displaying considerable toxicity [24,25,26]. Therefore, the development of TYR inhibitors with efficient bioactivity and less toxicity is a relevant branch of scientific research. Recently, we have used molecular modeling approaches to investigate biomolecular systems with an emphasis on enzymatic reactions and inhibition [27,28,29,30,31,32,33,34,35,36,37,38,39].

Herein, we report a computational study of analogs of KA in complex with TYR from *Bacillus megaterium* (TYR*Bm*). We also compared the binding of these inhibitors with l-Tyr and l-DOPA, where a dummy atom model [40] was used to represent Cu^2+^ ions in the active site of TYR*Bm*. This model provides a suitable description of structural and electrostatic effects by using “dummy” atoms around a metal ion and has been applied successfully on previous studies [41,42,43]. The binding free energies for the set of ligands were calculated with the linear interaction energy (LIE) approach [44]. Overall, this work shines a light on the inhibitor–tyrosinase complex interactions, which is paramount for the development of new inhibitors of TYR.

## 2. Results and Discussion

### 2.1. Molecular Docking Simulations

To evaluate the technical procedures used for molecular docking, a re-docking simulation was carried out using the atomic coordinates of natural substrates (l-Tyr and l-DOPA) and the KA inhibitor as an experimental reference. Our molecular re-docking results are summarized in Table 1. According to the re-docking procedure, all ligands bind into the active site of TYR and maintain key interactions with Cu^2+^ ions. Besides, the conformations obtained from molecular docking procedures are in good agreement with experimental data, demonstrated by small root-mean-square deviation (RMSD) values (< 0.50 Å) when superimposed with the experimental structures. The binding affinity values computed by the MOLDOCK scoring function appear to follow reality since l-Tyr and l-DOPA are natural substrates of TYR (excellent binding energies) while KA is a competitive inhibitor [45] with slightly weaker binding energy. Our molecular docking results are in agreement with previous in silico studies [46,47,48,49,50,51]. The 3D structures of the molecular docking results are available in Protein Data Bank (PDB) format in the Supplementary Materials.

**Figure 3:**
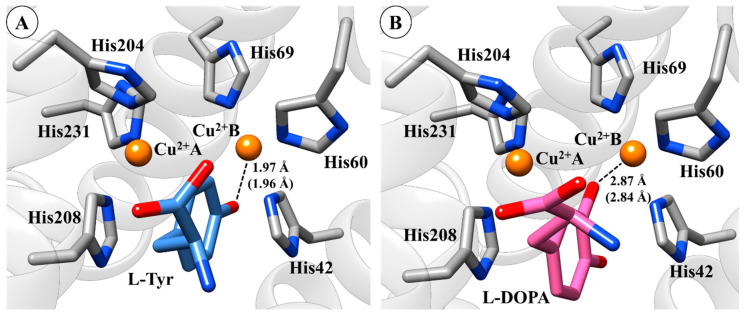
Binding mode of l-Tyrosine (l-Tyr) (**A**) and l-3,4-dihydroxyphenylalanine (l-DOPA) (**B**) in the active site of tyrosinase (TYR) obtained by re-docking. The 3D structures of the molecular docking results are available in Protein Data Bank (PDB) format in the Supplementary Materials (see Figure S1).

By performing a structural analysis of molecular docking results, some key interactions between groups of ligands and amino acid residues of TYR should be highlighted. It is worth mentioning that the hydroxyl group of l-Tyr ligand is positioned towards Cu^2+^B ion (distance 1.97 Å) and the aromatic ring of l-Tyr interacts with His208 through a π–π stacking interaction (Figure 3A). Note that these interactions are in agreement with experimental data [17,52,53], which demonstrate that the re-docking protocol used here was able to reproduce these key interactions in the active site of TYR. In the l-DOPA system, the re-docking results show that the phenol group of l-DOPA is also positioned towards a Cu^2+^B ion (distance 2.87 Å), which is also in agreement with previous studies (Figure 3B) [53,54,55].

**Figure 4:**
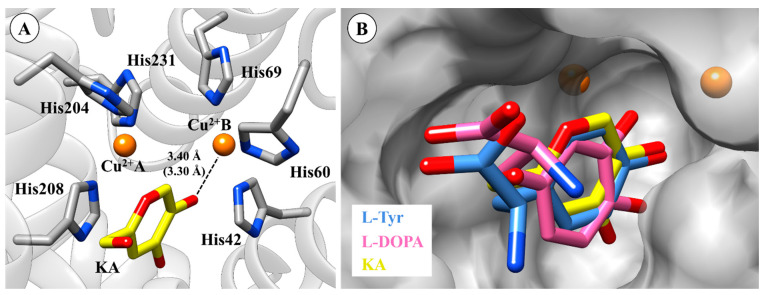
Binding mode of KA (kojic acid) (**A**) in the active site of tyrosinase (TYR) and (**B**) overlapping of l-Tyr, l-DOPA, and KA. Crystal (in parenthesis) and docking distances are reported in ångström (Å). The 3D structures of the molecular docking results are available in Protein Data Bank (PDB) format in the Supplementary Materials.

For the KA system, the inhibitor is stabilized by π–π stacking interaction in the active site of TYR (see Figure 4A), which agrees with a published crystal structure [45]. The oxygen atom of the hydroxyl group of KA is positioned towards the Cu^2+^B ion (distance 3.4 Å). Finally, the overlay between the l-Tyr, l-DOPA, and KA in the active site of TYR is depicted in Figure 4B.

According to the re-docking results above and previous studies [46,49,56], the docking protocol used can be confidently applied to describe TYR systems. Then, by using the same re-docking procedures, analogs of KA were submitted to the docking protocols. Our results suggest that KA analogs bind to the active site of TYR, acting as competitive inhibitors, since they display similar interactions as KA with the enzyme. Among the most important interactions is the interaction with a key residue His208 through π–π stacking interaction, which has been related to the binding of TYR inhibitors into the catalytic site [57]. To improve our understanding of the binding of TYR inhibitors, TRO [58], a potent inhibitor of TYR, was submitted to molecular docking analysis. For the TRO system, the docking results agree with the binding mode of this inhibitor complexed with TYR from *Agaricus bisporus* [59,60].

**Figure 5:**
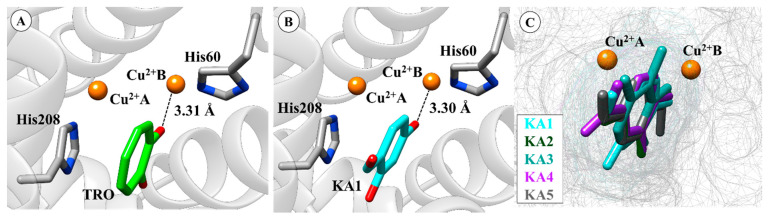
(**A**) Anchoring mode tropolone (TRO) on the active site of tyrosinase (TYR). (**B**) Anchoring mode KA1 on the active site of TYR. (**C**) Overlapping KA1, KA2, KA3, KA4 and KA5 on the active site. Docking distances are reported in ångström (Å). The 3D structures of the molecular docking results are available in Protein Data Bank (PDB) format in the Supplementary Materials (see Figure S2).

In particular, hydroxyl groups of TRO and KA1 interact with Cu^2+^B (Figure 5) producing distances from 3.31 Å and 3.30 Å, respectively. It can be seen that KA2 (3.30 Å), KA3 (3.67 Å), KA4 (3.33 Å) and KA5 (3.65 Å) also interact directly with Cu^2+^B. Among the most important interactions, we can highlight the interaction with a key residue His208 through π–π stacking interaction with all KA analogs.

Molecular docking results of KA analogs and TRO as inhibitors of TYR are summarized in Table 2.

### 2.2. Molecular Dynamics (MD) Simulation and CuDum Model

After molecular docking, 10 ns of classical MD simulations were performed for each system, where the CuDum model [40] was used for describing Cu^2+^ ions in the active site of TYR. Each copper ion is bridged by the imidazole rings of three His residues and three water molecules, so that a distorted octahedral geometry is observed (see Figure S3 in Supplementary Materials).

**Figure 6:**
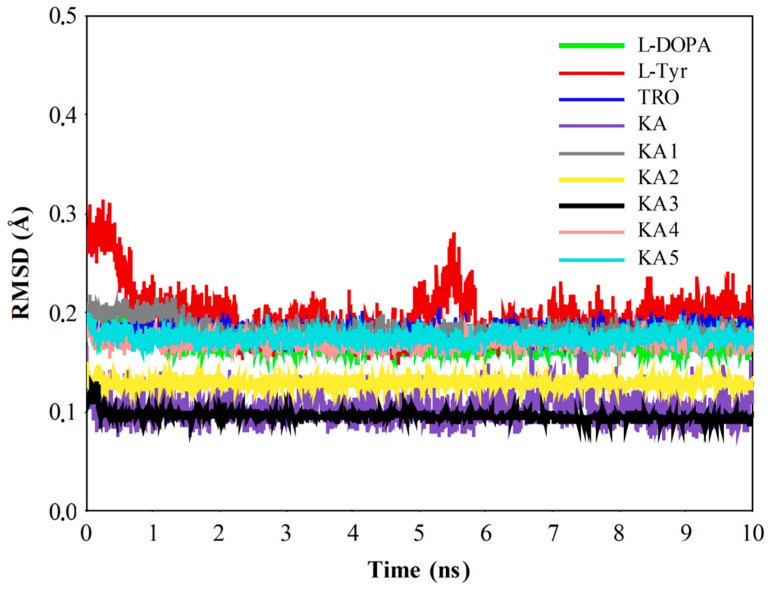
Root-mean-square deviation (RMSD) plot of tyrosinase (TYR) systems during 10 ns of molecular dynamic (MD) simulations.

According to the MD results, natural substrates (l-Tyr and l-DOPA), TRO, and KA and its analogs are stable in the catalytic site of TYR*Bm* (Figure 6), and interactions with copper ions and the amino acid residues constituting the active site are observed. Since the computed compounds have similar structures, they exhibit similar interactions with residues His42, His60, His204, Asn205, His208, and Val218, as presented in previous studies [47,48,50,51]. Thus, the molecular behavior of compounds interacting with the catalytic site of TYR is similar. Particularly, the l-Tyr system achieves structural stability after 6 ns of MD simulations, while other systems show stability during all 10 ns of MD.

As discussed previously, the CuDum model was applied to describe the Cu^2+^ ions in the catalytic site of TYR, where the introduction of dummy atoms surrounding the metal ion allows suitable capture of both structural and electrostatic effects (see details in Figure S3 in Supplementary Materials). Our MD results show the expected distance between the histidine residues present in the catalytic site and their respective Cu^2+^ ions (Table 3) in complexes with substrates (2.14 Å ± 0.03) and inhibitors (2.10 Å ± 0.03), which was also observed in previous studies [61]. According to Liao and co-workers, this classical Cu^2+^ model captures both the Jahn−Teller effect and maintains stable coordination geometries during MD simulations of metalloproteins without the need for artificial bonds between the metal center and the ligands. From our MD simulations, it was observed that the repulsion interaction between Cu^2+^A and Cu^2+^B ions are not excessive since the average atomic distance between these Cu^2+^ ions is only 0.70 Å higher than in a reported crystal structure [45]. Such a charge distribution in the dummy atom is particularly advantageous in systems with multiple metal centers, since the redistribution of charges reduces the excessive repulsion between metal sites [42].

**Figure 7:**
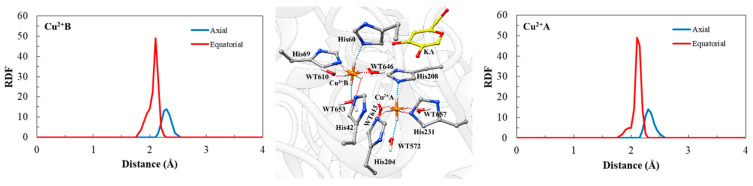
A representative snapshot of dummy models in TYR*Bm*-KA system taken from molecular dynamic (MD) simulations (on center). Radial distribution function around Cu^2+^A (right) and Cu^2+^B (left) for CuDum models into the catalytic site of TYR*Bm*-KA system. The enzyme portion is shown in the cartoon model, while the metal-binding sites are shown in the space-filling (CPK) model. Particularly, Cu^2+^ ion are in orange and their dummy atoms are in white. The 3D structure of this figure is available in Protein Data Bank (PDB) format in the Supplementary Materials.

Here, the CuDum model produces stable Cu^2+^ ion binding sites during the MD simulations of the TYR*Bm* systems (Figure 6). Furthermore, direct interactions between metal ions and substrates/inhibitors bound to the catalytic site of enzymes are observed. In the simulations of TYR*Bm* with the CuDum model, His208 residue prefers to interact with the axial dummy atom of Cu^2+^A, while the His204 and the His231 residues interact with the equatorial dummy atoms. For Cu^2+^B, the His60 residue prefers to interact with the axial dummy atom, while His42 and His69 residues interact with equatorial dummy atoms, which agrees with findings from the experiment [62,63,64,65] and other quantum-mechanics-based calculations [66,67,68] (Figure 7).

Throughout the MD simulations, the coordination geometry remains a distorted octahedral for the CuDum model with the His residues, and the bond distances are quite close to those in the crystal structure (PDB ID: 5138) [45] (Table 3). The coordination of water molecules to CuDum is in good agreement with an experimental finding that the aqueous involvement [69] is necessary for the oxidation catalytic activity at the metal-binding site of the TYR enzyme [1,16]. Therefore, the applied CuDum model captures the Jahn–Teller effect occurring in the catalytic site of the TYR system and stable coordination geometries are maintained during the MD simulations with no artificial bonds being observed between the metal centers and their residue-ligands.

### 2.3. Linear Interaction Energy (LIE)

The thermodynamic knowledge of the binding affinity between a protein–inhibitor system is key to a better understanding of molecular recognition. Therefore, an accurate and efficient method to compute binding free energy (ΔG_bind_) is essential in computer-aided drug design [70]. A set of ΔG_bind_ methods is available, from rigorous methods such as thermodynamic integration (TI) [71] and free energy perturbation (FEP) [72] as well as simple empirical scoring functions computed in molecular docking [73]. The first methods are computationally more expensive, since they demand extensive sampling of multiple intermediate nonphysical states, making them unsuitable for application in high-throughput scenarios [74]. On the other hand, the empirical scoring functions are good for predicting binding poses in protein–ligand systems, even for large chemical databases [75], but they usually fail in quantitatively predicting and ranking ΔG_bind_ values [76]. Therefore, a suitable alternative in terms of effectiveness and efficiency to compute ΔG_bind_ is by taking into account the configurational space for the ligand, protein–ligand, and solvent in the protein–ligand bound and unbound states only [77]. These features offer advantages for both rigorous free energy methods (in terms of efficiency) and empirical scoring functions (in terms of accuracy) [78,79]. Among the so-called end-point methods, we have applied in this study the linear interaction energy (LIE) approach [44].

As discussed above, the binding affinity of inhibitors towards enzymes is key for the development of new drug candidates [80]. Here, we applied the LIE approach [44] to describe the binding free energy values of the ligands (natural substrates and inhibitors). The method was developed as an alternative to more time-consuming free energy perturbation calculations [78,79,81,82,83]. Several research groups have reported LIE calculations on various systems using different programs, force fields, and computational procedures [49,84,85,86].

Here, the last 4 ns of MD simulations for each system were used to compute binding free energy values. The results for the binding affinity of the ligands computed through LIE are depicted in Table 4. The empirical parameters α and β, used directly in the calculation of the binding affinity, were obtained in two ways to serve as a point of comparison and thus perceive which parameters best represent the system with the metal protein TYR. In the first linear regression model, the parameters were obtained directly from the literature (α = 0.181 e β = 0.5, 0.43, 0.37 and 0.33) [87] and for the second model, the parameters (α = 0.197 and β = 0.342) were obtained from the linear fitting with experimental binding free energy, where, in both models, the Jahn–Teller effect is taking into account that the CuDum model was used in all MD systems for the bound models. Here, the ΔG_LIE1_ refers to binding energy values computed by α and β parameters from literature, while ΔG_LIE2_ values are computed by including α and β from the linear fitting procedure. The values of α and β used for each inhibitor in the ΔG_LIE1_ model are described in Table S1 of the Supplementary Materials.

The values of binding affinity, derived from molecular dynamics (MD) trajectories from the LIE method are in good agreement with the experimental data reported in previous studies [45,49,58,88,89]. To provide more reliable results and to enhance the robustness of the approach used for the prediction of the binding free energy of the compounds studied, we applied α and β parameters from the linear fitting between polar and non-polar terms and experimental binding free energy, which was satisfactory. Particularly, the absolute binding energies calculated for L-Tyr, TRO, and KA using LIE (models 1 and 2) agree with the experimental values (−9.50, −8.30, and −7.46 kcal/mol, respectively) [45,58]. Although LIE is an approximation method, these results provide good confidence regarding the efficacy of the applied procedures.

In general, the theoretical binding affinities of KA analogs from the MD trajectories through the LIE method are in excellent agreement with experimental data (Table 4). The KA5 inhibitor showed the weakest binding affinity with the TYR*Bm* enzyme (also according to experiment, ΔG_EXP_ = −4.68 kcal/mol), the reason for this weaker affinity was not revealed in the experimental paper [89]; however, our MD simulations suggest that absence of the hydroxyl group can be responsible for its weak binding.

**Figure 8:**
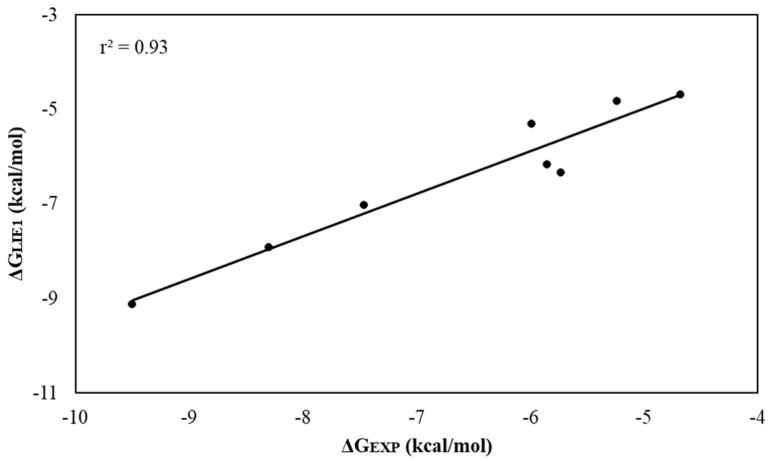
Linear regression graph between ΔG_LIE1_ and ΔG_EXP_. Values in kcal/mol.

The coefficients of determination (*r*^2^) obtained for the linear regression of predicted values versus the observed contributions were equal to 0.93 and 0.94 for LIE model 1 (Figure 8) and model 2, respectively. These results suggest that the standard parameterization (model 1) and that obtained from the linear fitting procedure (model 2) (Figure S4) of LIE are both robust methods that reproduce the experimental affinities of TYR inhibitors.

### 2.4. Residual Decomposition Analysis

**Figure 9:**
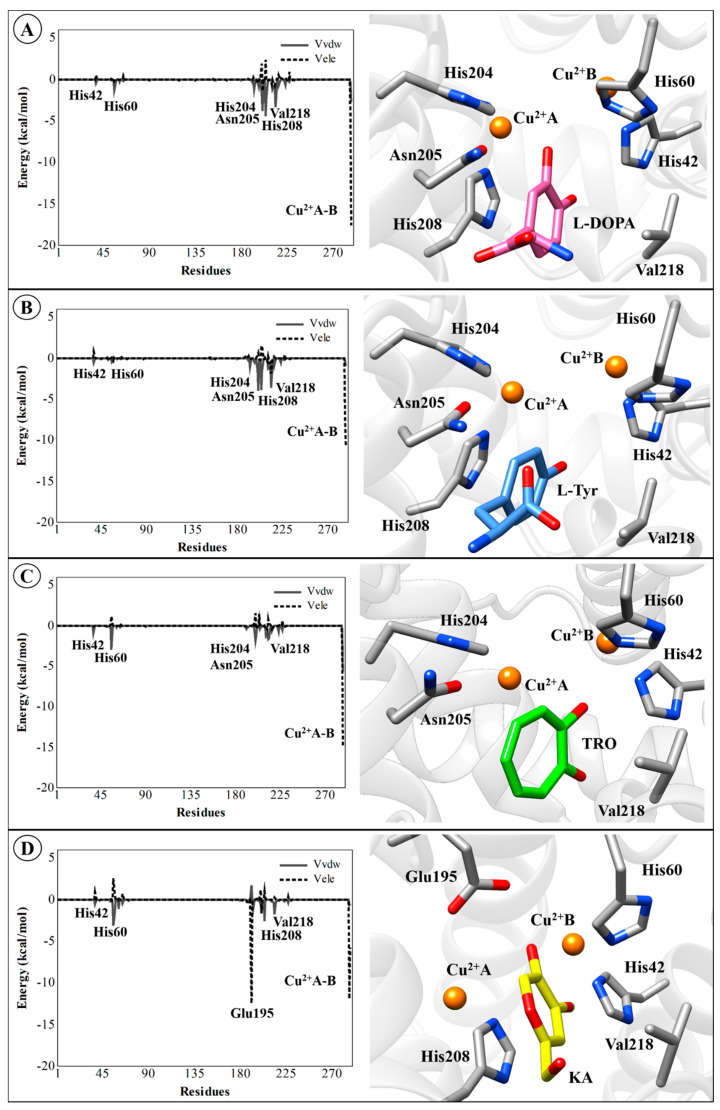
Average ligand–residue interaction energies (in kcal/mol) overall in compounds used in the linear interaction energy (LIE) calculations for the residues that contribute most to the ligand-surrounding electrostatic (dashed line) and (solid line) Van der Waals. (**A**) TYR*Bm*-l-DOPA, (**B**) TYR*Bm*-l-Tyr, (**C**) TYR*Bm*-TRO, (**D**) TYR*Bm*-KA. The 3D structures of this figure are available in Protein Data Bank (PDB) format in the Supplementary Materials Materials (see Figure S5).

**Figure 10:**
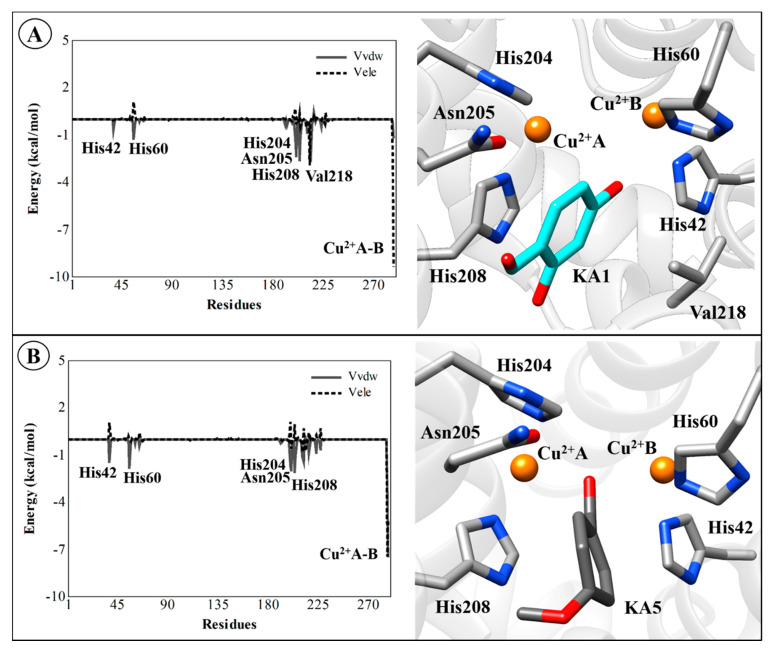
Average ligand–residue interaction energies (in kcal/mol) overall in compounds used in the linear interaction energy (LIE) calculations for the residues that contribute most to the ligand-surrounding electrostatic (dashed line) and (solid line) van der Waals. (**A**) TYR*Bm*-KA1 and (**B**) TYR*Bm*-KA5. The 3D structures of this figure are available in Protein Data Bank (PDB) format in the Supplementary Materials.

Although there is no rigorous way to decompose free energies into contributions into protein–ligand systems from the MD simulations [90,91], the electrostatic and van der Waals interaction energies of the TYR–ligand complexes can be computed to identify which residues are important for binding. Therefore, the average electrostatic and van der Waals interaction energies for all compounds were evaluated. These interactions for natural substrates (l-DOPA and l-Tyr), inhibitors (TRO and KA), the most active (KA1) and the less (KA5) active analog for the TYR residues (including Cu^2+^ ions) are presented in Figure 9 and Figure 10. The Cu^2+^A and Cu^2+^B ions make the largest contributions to the electrostatic component of the average ligand–residue interactions, as can be seen in Table 5. However, particularly for the TYR*Bm*–KA complex, the Glu195 residue makes considerable electrostatic contributions (−12.45 kcal/mol) to the ligand–enzyme interaction energy through a hydrogen interaction between the carboxylic acid group of amino-acid residue and the hydroxyl group of KA.

Interestingly, compounds that have hydroxyl groups exhibit interaction with one of the Cu^2+^ ions (A or B). Particularly, KA5 interacts with the Cu^2+^ ions through its aldehyde group. However, this interaction is not observed for the other compounds. These results are in concordance with previous studies that recognize molecules containing hydroxyl groups as potential TYR inhibitors [49,92,93,94]. As can be observed in Table 4, ΔV_ele_ and ΔV_vdW_ terms vary widely among these ligands. Particularly, the electrostatic contributions from Cu^2+^ ions implies that the ΔV_ele_ term has a strong influence on the binding affinity of ligands. Structurally, these energetic contributions can be traced back to the strength of the interaction between the Cu^2+^ ion and the hydroxyl group of the ligands, except for the KA5, which interacts through an aldehyde group.

The non-polar free energy contribution, calculated from the change in ligand-surrounding van der Waals energy, shows some residues present in the catalytic site of TYR, such as His204, Asn205, His208, and Val218, which are important for the non-polar interactions (van der Waals) with natural substrates l-DOPA (Figure 9A) and l-Tyr (Figure 9B), with average values of −1.54, −3.86, −4.12, and −3.45 kcal/mol, respectively. For other systems, van der Waals interactions have suitable contributions (see Figure S4 in Supplementary Materials). However, it should be noted that these interactions are not as easily interpreted as the polar term in the LIE method because they do not only take into account the van der Waals interactions but also size-dependent terms such as the hydrophobic effect [44,95]. It is worth mentioning that, in general, the non-polar contribution (van der Waals) is less expressive than the polar (electrostatic) contribution. The binding affinity is favored by polar interactions, mainly from the copper ions present in the active site. Therefore, the obtained correlation between activity and only a few interaction energies suggests that efficient LIE models may be based on only monitoring a subset of the ligand–residue interactions. All values of polar and non-polar contributions for all TYR systems are detailed in Tables S2 and S3 in Supplementary Materials.

## 3. Materials and Methods

### 3.1. Molecular Docking Simulations

The initial atomic coordinates for the simulations were extracted from the Protein Data Bank (PDB) under codes 5I38 [45], 4P6R [53], and 4P6S [53], which are TYR from *Bacillus* *megaterium* (TYR*Bm*) with KA, l-Tyr, and l-DOPA in the active site, respectively. In this stage, copper ions were described only as van der Waals spheres at the active site of TYR. The molecular docking simulations were performed using Molegro Virtual Docker (MVD) version 5.5 [73], which has been applied successfully for TYR systems [46,49,56]. In this software, the search algorithm named as MOLDOCK is applied by computing a heuristic search algorithm, where a differential evolution and a cavity prediction algorithm are taken into account [96]. The MOLDOCK, which is an extension of the piecewise linear potential (PLP), includes new hydrogen bonding and electrostatic contributions. A re-ranking scoring function is also computed to select the most promising docking result among those found by the docking algorithm. The re-docking procedure shows that the algorithm used was able to predict the binding mode of KA, l-Tyr, and l-DOPA in the active site of TYR. The MOLDOCK scoring function (Equation (1)) [96] was used to rank the potential ligand poses.

(1) $$E_{score}=E_{inter}+E_{intra}$$

The MOLDOCK scoring function was set with a grid resolution of 0.30 Å. It was set at a maximum iteration of 3000 with a simplex evolution size of 100. The best pose with a smaller RMSD value was selected for subsequent ligand–protein interaction energy analysis. The score for ligand–protein non-bonding interactions was obtained through:

(2) $$E_{inter}=\overset{N}{\underset{i=l}{\sum }}\overset{M}{\underset{j=l}{\sum }}\left(E_{PLP}\left(r_{ij}\right)+332\frac{q_{i}q_{j}}{r_{ij}}\right)$$

where $E_{PLP}$ is the piecewise linear potential and $q_{i}$ and $q_{j}$ correspond to the charges of *i* and *j* atoms, respectively. Finally, $r_{ij}$ is the interatomic distance between *i* and *j* atoms. Intramolecular interactions are obtained through:

(3) $$E_{intra}=\underset{i\in lig}{\sum }\underset{j\in lig}{\sum }E_{PLP}\left(rij\right)+\underset{ligflexible}{\sum }A$$

where θ is the torsion and $E_{clash}$ is the penalty attributed when the distance between two atoms is lower than 2 Å.

Finally, KA analogs were built by using MarvinSketch version 17.27 [97] and structure optimizations at PM6 level [98] were carried out in Gaussian09 [99]. All compounds studied here are depicted in Table 6. It is worth noting that during the molecular docking, Cu^2+^ ions in the active site of TYR were treated as Van der Waals sphere, where electrostatic interaction was neglected.

### 3.2. Molecular Dynamics (MD) Simulations

The best-ranked structures obtained from molecular docking results were selected as starting structures for the MD simulations. In this stage, Cu^2+^ ions present in the active site of TYR*Bm* were treated by using the Cu^2+^ dummy model (CuDum) proposed by Liao and coworkers [40]. The MD simulations were performed using the Q program [100]. The OPLS-AA force field [101] and TIP3P water model [102] were selected to describe the solute (enzyme and ligands) and solvent (water), respectively. Particularly, classical parameters for the ligands (natural substrates and inhibitors) were obtained through an automatic parameterization carried out by MACROMODEL [103]. Initially, pKa values of all titratable residues were computed by using the PROPKA method [104] as implemented in the PDB2PQR server [105] at neutral pH.

Then, all systems were solvated with a 20 Å radius simulation sphere of the TIP3P water model [102] centered on the center of mass of the respective ligand. The simulation sphere was subjected to polarization and radial constraints by applying the surface constrained all-atom solvent (SCAAS) model [106] at the sphere surface, to describe appropriately the properties of bulk water. Titratable residues close to the sphere boundary were modeled in their neutral form to account for dielectric screening [100].

Afterwards, each system was submitted to the following MD simulation phases: (a) heating, (b) equilibration, and (c) production, which was selected for data collection. Non-bonded interaction energies were computed by applying a 10 Å cutoff, excluding only the ligand atoms. Long-range electrostatic interactions were computed through the local reaction field (LRF) multiple expansion approach [107]. The non-bonded pair lists and the ligand-surrounding interaction energy values were saved every 25 steps. To save computational resources, all atoms outside of the 20 Å radius simulation sphere were restrained to their initial positions [100].

Initially, 5000 steps of MD simulations were applied using a very short time step (equal to 0.1 fs) and T = 1K, coupled to a strong bath (0.1 fs bath coupling), where heavy atoms were restrained by applying a force constant equal to 50 kcal/mol∙Å^2^. After, each system was gradually heated to 300 K over 50 ps with time step and the bath coupling equals to 1 fs and 100 fs, respectively. The restrain force constant applied on all solute-heavy atoms was gradually removed.

Finally, for each system, 10 nanoseconds (ns) of MD simulations at 300 K were performed out for the data collection. The time step was set to 2 fs, and all bonds involving hydrogen atoms were restrained applying the SHAKE [108] algorithm. Particularly, for the water (free state) simulations, ligands were kept to the center of their water simulation sphere by using a weak harmonic restraint to the center of mass of the ligands.

### 3.3. Linear Interaction Energy (LIE) Approach

MD results obtained in the previous section in combination with the LIE approach [44] were used to calculate the binding affinities of KA and its analogs into the TYR*Bm* enzyme. The LIE method uses the ensembles of the free ligand in solution and the protein–ligand complex to calculate the change in free energy associated with binding to the protein [44]. Then, the difference in interaction energies between them is used to calculate the free binding energy, according to Equation (4):

(4) $$\Delta G_{LIE}=\alpha \left(\langle V_{vdw}\rangle {}_{bound}-\langle V_{vdw}\rangle {}_{free}\right)+\beta \left(\langle V_{ele}\rangle {}_{bound}-\langle V_{ele}\rangle {}_{free}\right)$$

where the α parameter is the empirically derived non-polar scaling factor and β is the scaling factor of the polar contribution dependent on the chemical nature of the ligand [87]. The term $\langle V_{vdw}\rangle$ represents the average of the ligand surrounding van der Waals (water plus protein in the “bound” case and only water in the “free” term subscript). The term $\langle V_{ele}\rangle$ represents the average for electrostatic interaction energies from the simulations of bound and free ligand [44].

Particularly, the LIE parameters (α and β terms) are typically obtained from the literature (α = 0.181 and β = 0.33−0.50) [87] and obtained by linear fitting the ligand-surrounding interaction energies versus experimental binding affinities.

The experimental binding affinity values were calculated using Equation (5):

(5) $$\Delta G_{bind}^{exp}=RTlnK_{i}$$

The experimental *K_i_* values were obtained from literature [45,49,58,88,89].

## 4. Conclusions

We investigated the accuracy of molecular docking and MD simulations in combination with the LIE method on a set of TYR inhibitors. Particularly, during MD simulations, a dummy model including the description of the Jahn–Teller effect for Cu^2+^ ions in the active site of the TYR enzyme was included. The standard form and parameterization of the LIE method accurately reflect the binding activities of compounds in the catalytic site of TYR. The TYR–ligand interactions were analyzed in detail, and it was found that the binding free energies of the studied set of TYR inhibitors and their natural substrates can be described mainly by electrostatic contributions of Cu^2+^ ions present in the catalytic site of the enzyme. Binding free energy results computed with the LIE method, using standards and specific scaling parameters for the LIE equation, also agree very well with experimental binding values. Thus, a combination of computational techniques may facilitate the understanding of potential TYR inhibitors and the prediction of main features for improvement of their inhibitory activity.

## Figures and Tables

**Table 1:** MOLDOCK scoring (in kcal/mol) and RMSD (in Å) values for re-docking procedures.

Compound	MOLDOCK	RMSD
l-Tyr	−89.77	0.22
l-DOPA	−90.25	0.29
KA	−69.26	0.31

l-Tyr: l-Tyrosine; l-DOPA: l-3,4-dihydroxyphenylalanine; KA: kojic acid

**Table 2:** MOLDOCK scoring values (in kcal/mol) of tyrosinase (TYR) inhibitors.

Inhibitor	MOLDOCK
TRO	−72.31
KA	−69.26
KA1	−68.72
KA2	−67.26
KA3	−62.76
KA4	−65.45
KA5	−55.47

TRO: Tropolone; KA: Kojic acid.

**Table 3:** Experimental and average simulated distances (in Å) between His residues (or water molecules) and copper ions (Cu^2+^A and Cu^2+^B) in the TYR*Bm*-KA system. The experimental distances for His residues were taken from the crystal structure (Protein Data Bank (PDB) code: 5I38) [45]. For the water molecules, the experimental values are 1.96 and 2.28 Å for water molecules in the equatorial and axial positions, respectively [69].

Residue (Atom)	Ion	Experimental Distance	Average Simulated Distances
His204 (NE2)	Cu^2+^A	2.09	2.15 ± 0.07
His208 (NE2)		2.07	2.30 ± 0.07
His231 (NE2)		2.02	2.02 ± 0.11
WT572 (O)		2.28	2.34 ± 0.07
WT613 (O)		1.96	2.11 ± 0.04
WT657 (O)		1.96	2.12 ± 0.03
His42 (NE2)	Cu^2+^B	2.09	2.00 ± 0.09
His60 (NE2)		2.07	2.33 ± 0.06
His69 (NE2)		2.02	2.01 ± 0.10
WT610 (O)		1.96	2.11 ± 0.03
WT646 (O)		1.96	2.12 ± 0.03
WT653 (O)		2.28	2.26 ± 0.06

His: Histidine; WT: Water; NE2: Nitrogen atom; O: Oxigen atom; Cu^2+^: Copper ion.

**Table 4:** Theoretical and experimental binding free energy values (in kcal/mol) calculated for each tyrosinase (TYR) system.

Molecule	ΔV_vdW_	ΔV_ele_	ΔG_LIE1_	ΔG_LIE2_	ΔG_EXP_
l-DOPA	−17.95 ± 0.53	−22.93 ± 1.02	−10.79 ± 0.32	−11.20 ± 0.33	
l-Tyr	−12.44 ± 0.37	−20.91 ± 0.82	−9.13 ± 0.24	−9.47 ± 0.22	−9.50
TRO	−12.9 ± 0.19	−15.16 ± 0.97	−7.93 ± 0.53	−7.60 ± 0.48	−8.30
KA	−5.68 ± 0.08	−18.24 ± 0.26	−7.04 ± 0.27	−7.28 ± 0.28	−7.46
KA1	−8.76 ± 0.30	−11.32 ± 0.53	−5.31 ± 0.24	−5.51 ± 0.25	−5.99
KA2	−12.91 ± 0.03	−11.68 ± 0.12	−6.17 ± 0.15	−6.42 ± 0.15	−5.85
KA3	−6.09 ± 0.44	−14.19 ± 0.18	−6.34 ± 0.80	−5.98 ± 0.72	−5.73
KA4	−16.68 ± 0.05	−5.56 ± 0.10	−4.83 ± 0.66	−5.05 ± 0.68	−5.24
KA5	−17.14 ± 0.10	−3.75 ± 0.08	−4.69 ± 0.03	−4.53 ± 0.02	−4.68

l-DOPA: l-3,4-dihydroxyphenylalanine; l-Tyr: l-Tyrosine; TRO: Tropolone; KA: Kojic acid; vdW: Van der Waals; ele: Electrostatic; LIE: Linear interaction energy; EXP: Experimental

**Table 5:** Electrostatic contributions (V_ele_, in kcal/mol) of Cu^2+^A and Cu^2+^B ions calculated by the linear interaction energy (LIE) method.

Complex	Dummy Atom	Distance (Ligand—Cu^2+^)	V_ele_
TYR*Bm—*l-DOPA	Cu^2+^A	2.79	−17.63
	Cu^2+^B	4.45	−4.16
TYR*Bm—*l-Tyr	Cu^2+^A	3.75	−10.81
	Cu^2+^B	4.69	−7.39
TYR*Bm—*TRO	Cu^2+^A	2.21	−15.04
	Cu^2+^B	4.73	−1.98
TYR*Bm—*KA	Cu^2+^A	5.27	−5.70
	Cu^2+^B	4.82	−11.88
TYR*Bm—*KA1	Cu^2+^A	4.56	−9.37
	Cu^2+^B	5.46	−2.26
TYR*Bm—*KA2	Cu^2+^A	3.09	−8.59
	Cu^2+^B	3.76	−1.42
TYR*Bm—*KA3	Cu^2+^A	3.71	−9.91
	Cu^2+^B	3.68	−7.77
TYR*Bm—*KA4	Cu^2+^A	3.68	−2.50
	Cu^2+^B	3.46	−1.07
TYR*Bm—*KA5	Cu^2+^A	3.59	−5.22
	Cu^2+^B	3.64	−7.47

**Table 6:** Experimental inhibition (K_i_) and dissociation (K_d_) constants for Tyrosinase (TYR) ligands.

Molecules	Nomenclature IUPAC	Structure 2D	K_i_ (K_d_)
l-DOPA	(2S)-2-amino-3-(3,4-dihydroxyphenyl)propanoic acid	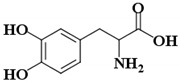	-
l-Tyr	(2S)-2-amino-3-(4-hydroxyphenyl)propanoic acid	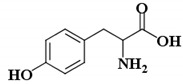	* (0.1 µM) [45]
TRO	2-Hydroxy-2,4,6-cycloheptatrien-1-one	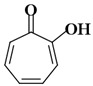	0.8 µM [58]
KA	5-Hydroxy-2-(hydroxymethyl)-4H-pyran-4-one	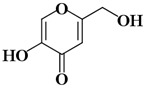	3.5 µM [45]
KA1	2,4-dihydroxybenzaldehyde	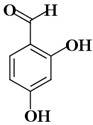	41.55 µM [88]
KA2	3,4-dihydroxybenzaldehyde	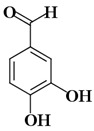	52.96 µM [88]
KA3	3-hydroxy-1,2-dimethyl-4(1h)-pyridone	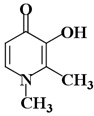	64 μM [49]
KA4	5-hydroxy-4-oxo-4h-pyran-2-carboxylic acid	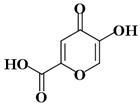	145 μM [49]
KA5	4-Methoxybenzaldehyde	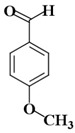	376 μM [89]

l-DOPA: l-3,4-dihydroxyphenylalanine; l-Tyr: l-Tyrosine; TRO: Tropolone; KA: Kojic acid. * K_d_ value (dissociation constant) for the substrates.

## Supplementary Materials

Supplementary materials can be found at https://www.mdpi.com/1422-0067/21/13/4783/s1. Figure S1: Theoretical binding mode of (A) KA2 and (B) KA3 compounds on the active site of TYR; Figure S2: Theoretical binding mode of (A) KA4 and (B) KA5 compounds on the active site of TYR; Figure S3: 3D representation of the CuDum model. The structural and energetic effects of the Cu^2+^ ion is computed by the point charge of the metal ion which is distributed to six “dummy” atoms with partial charge δ+ on axial and equatorial positions; Figure S4: Linear regression graph between ΔG_LIE2_ and ΔG_EXP_. Values in kcal/mol; Figure S5: Average ligand–residue interaction energies (in kcal/mol) overall in compounds used in the LIE calculations for the residues that contribute most to the ligand-surrounding electrostatic (dashed line) and (solid line) van der Waals. (a) TYRBm-KA2, (b) TYRBm-KA3, (c) TYRBm-KA4; Table S1: Empirical LIE parameters used on ΔGLIE1 model; Table S2: Individual polar and non-polar residual contributions of L-DOPA, L-Tyr, TRO, and KA systems. The values are reported in the kcal/mol; Table S3: Individual polar and non-polar residual contributions of KA analogs systems. The values are reported in the kcal/mol.
