# Supplementary material for: Evaluating the Performance of a Non-Bonded Cu2+ Model Including Jahn−Teller Effect into the Binding of Tyrosinase Inhibitors
Source: Int J Mol Sci. 2020 Jul 6;21(13):4783. doi: 10.3390/ijms21134783 (PMC7369908; doi:10.3390/ijms21134783)
Supplement: Supplementary file 1 [file ijms-21-04783-s001.zip › ijms-832895 - Supplementary - final.pdf]

Supplementary Materials

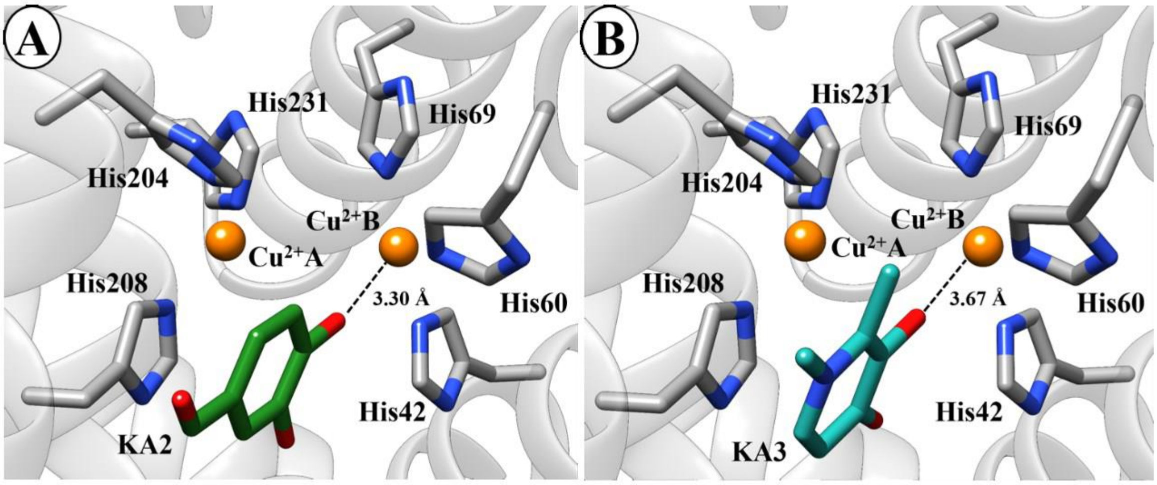

Figure S1. Theoretical binding mode of (**A**) KA2 and (**B**) KA3 compounds on the active site of TYR.

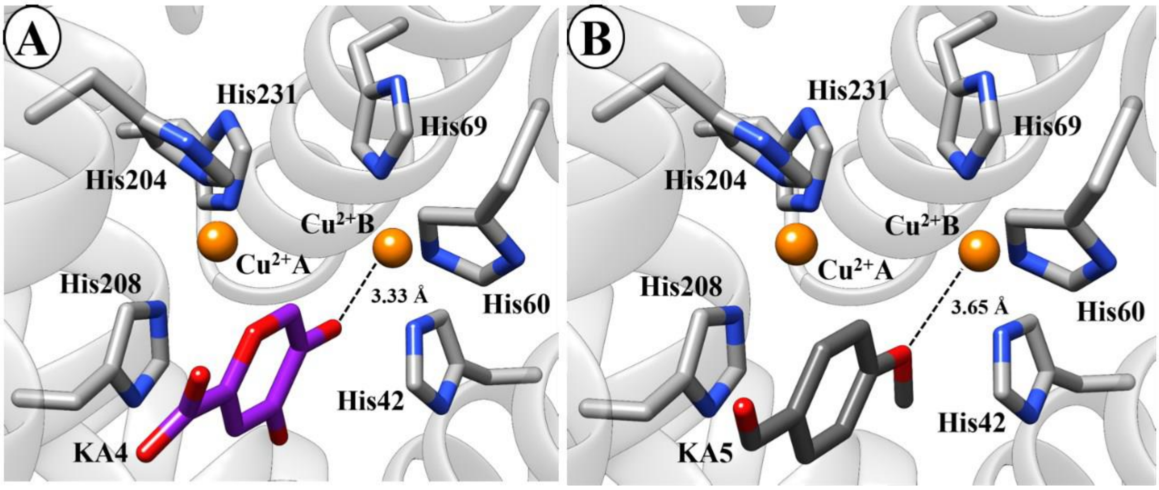

Figure S2. Theoretical binding mode of (**A**) KA4 and (**B**) KA5 compounds on the active site of TYR.

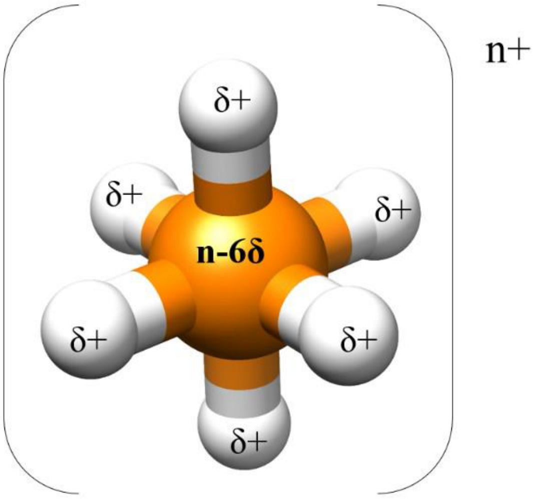

Figure S3. 3D representation of the CuDum model. The structural and energetic effects of Cu^{2+} ion is computed by the point charge of the metal ion which is distributed to six "dummy" atoms with partial charge δ+ on axial and equatorial positions.

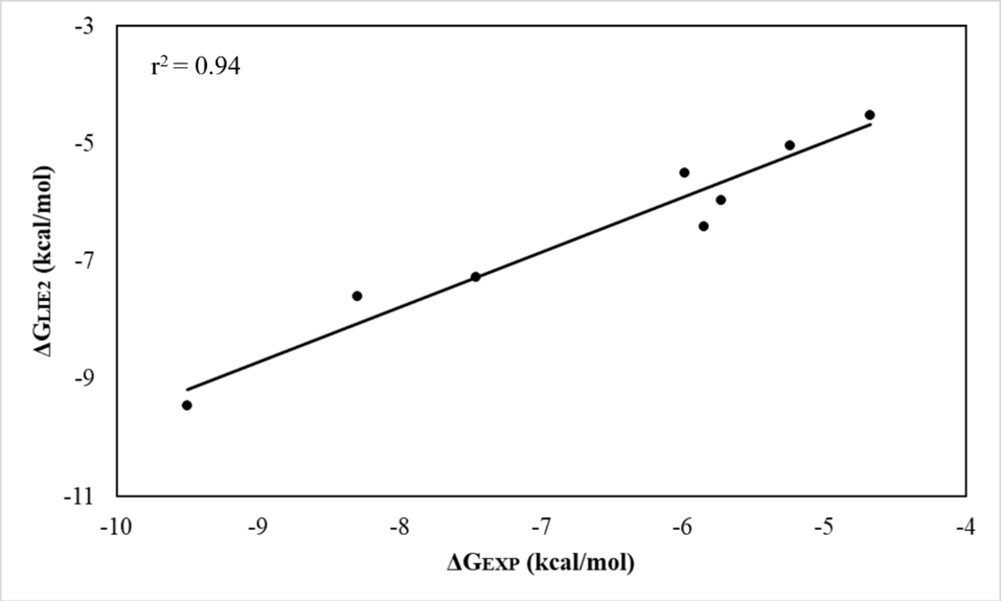

Figure S4. Linear regression graph between ΔG_{LIE2} and ΔG_{EXP}. Values in kcal/mol.

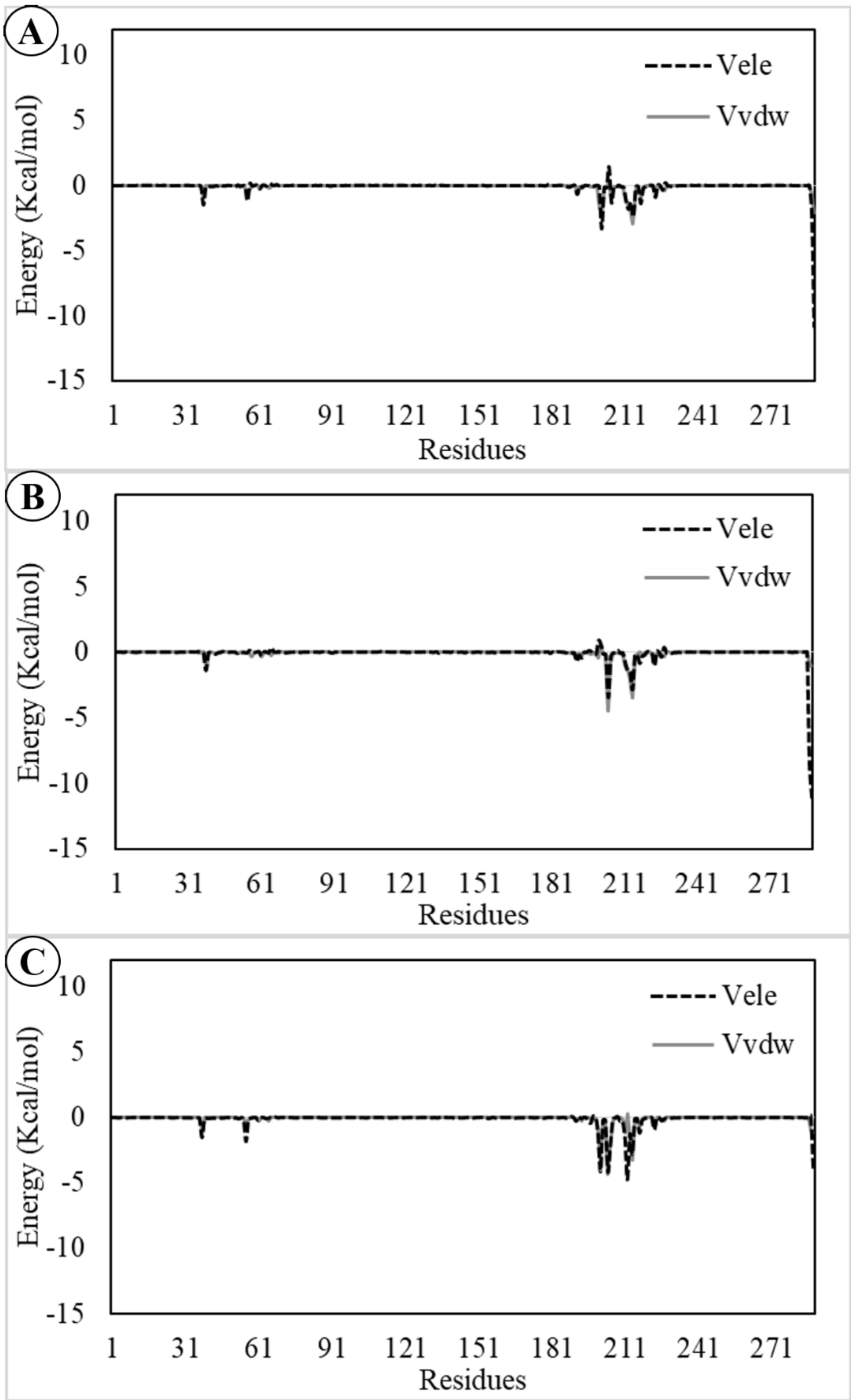

Figure S5. Average ligand-residue interaction energies (in kcal/mol) overall compounds used in the LIE calculations for the residues that contribute most to the ligand-surrounding electrostatic (dashed line) and (solid line) van der Waals. (**a**) TYR*Bm*-KA2, (**b**) TYR*Bm*-KA3, (**c**) TYR*Bm*-KA4.

Table S1. Empirical LIE parameters used on ΔG_{LIE1} model.

Inhibitor	ΔGLIE1	
	α	β
L-DOPA	0.18	0.33
L-Tyr	0.18	0.33
TRO	0.18	0.37
AK	0.18	0.33
KA1	0.18	0.33
KA2	0.18	0.33
KA3	0.18	0.37
KA4	0.18	0.33
KA5	0.18	0.43

Table S2. Individual polar and non-polar residual contributions of L-DOPA, L-Tyr, TRO and KA systems. The values are reported in the kcal/mol.

Residue	L-DOPA		L-Tyr		TRO		KA	
	V_{vdw}	V_{ele}	V_{vdw}	V_{ele}	V_{vdw}	V_{ele}	V_{vdw}	V_{ele}
Tyr5	0.00	0.00	0.00	0.00	0.00	0.00	0.00	0.00
Arg6	0.00	−0.01	0.00	−0.01	0.00	−0.01	0.00	−0.01
Val7	0.00	0.00	0.00	0.00	0.00	0.00	0.00	0.00
Arg8	0.00	−0.03	0.00	−0.02	0.00	−0.01	0.00	0.00
Lys9	0.00	0.01	0.00	0.02	0.00	0.01	0.00	0.01
Asn10	0.00	−0.01	0.00	−0.01	0.00	0.00	0.00	0.01
Val11	0.00	−0.02	0.00	−0.02	0.00	0.00	0.00	0.00
Leu12	0.00	−0.01	0.00	−0.01	0.00	0.00	0.00	0.00
His13	0.00	−0.01	0.00	−0.02	0.00	−0.01	0.00	−0.01
Leu14	0.00	0.00	0.00	−0.01	0.00	−0.01	0.00	0.00
Thr15	0.00	0.01	0.00	0.01	0.00	0.01	0.00	0.01
Asp16	0.00	0.00	0.00	0.00	0.00	0.00	0.00	0.00
Thr17	0.00	0.00	0.00	0.00	0.00	0.00	0.00	0.00
Glu18	0.00	0.00	0.00	0.01	0.00	0.01	0.00	0.01
Lys19	0.00	0.00	0.00	0.00	0.00	0.00	0.00	0.01
Arg20	0.00	0.00	0.00	0.00	0.00	0.00	0.00	0.01
Asp21	0.00	0.00	0.00	0.00	0.00	0.00	0.00	0.00
Phe22	0.00	0.00	0.00	0.00	0.00	0.00	0.00	0.01
Val23	0.00	0.00	0.00	0.00	0.00	0.01	0.00	0.01
Arg24	0.00	−0.01	0.00	0.00	0.00	0.00	0.00	0.00
Thr25	0.00	−0.01	0.00	0.00	0.00	0.00	0.00	0.00
Val26	0.00	−0.01	0.00	−0.01	0.00	0.00	0.00	0.00
Leu27	0.00	−0.01	0.00	0.00	0.00	0.00	0.00	0.00
Ile28	0.00	−0.01	0.00	−0.01	0.00	0.00	0.00	0.00
Leu29	0.00	−0.01	0.00	−0.01	0.00	0.00	0.00	0.00
Lys30	0.00	−0.02	0.00	−0.01	0.00	−0.01	0.00	−0.01
Glu31	0.00	−0.01	0.00	−0.01	0.00	0.00	0.00	0.00
Lys32	0.00	−0.01	0.00	−0.01	0.00	−0.01	0.00	−0.01
Gly33	0.00	0.00	0.00	0.00	0.00	0.00	0.00	0.00
Ile34	0.00	0.00	0.00	0.01	0.00	0.01	0.00	0.02
Tyr35	−0.01	0.03	0.00	0.03	−0.01	0.01	0.00	0.01
Asp36	0.00	−0.01	0.00	0.00	0.00	0.01	0.00	0.02
Arg37	0.00	−0.03	0.00	−0.01	0.00	0.00	0.00	0.01
Tyr38	−0.01	0.01	−0.01	0.03	−0.01	0.00	−0.01	0.04
Ile39	−0.02	−0.02	−0.02	0.02	−0.02	0.02	−0.01	0.05
Ala40	−0.01	−0.04	0.00	0.00	0.00	0.01	0.00	0.03
Trp41	−0.01	−0.04	−0.01	0.00	−0.01	−0.02	−0.01	0.01
His42	−0.46	0.58	−0.59	0.83	−0.87	−0.07	−0.54	0.95
Gly43	−0.02	−0.02	−0.02	0.06	−0.02	−0.03	−0.02	0.03
Ala44	−0.01	−0.04	−0.01	−0.01	−0.01	−0.03	−0.01	−0.01

Ala45	−0.02	−0.02	−0.01	−0.01	−0.01	−0.04	−0.02	−0.10
Gly46	−0.03	0.02	−0.03	0.02	−0.03	−0.02	−0.05	−0.10
Lys47	−0.01	−0.02	−0.01	−0.02	−0.01	−0.02	−0.02	−0.02
Phe48	−0.01	0.00	−0.01	0.02	−0.01	−0.01	−0.01	−0.02
His49	−0.01	0.01	−0.01	−0.08	−0.01	0.04	−0.01	0.05
Thr50	0.00	0.00	0.00	0.04	0.00	−0.01	−0.01	−0.01
Pro51	0.00	−0.02	0.00	0.01	0.00	−0.01	0.00	−0.01
Pro52	0.00	0.02	0.00	0.00	0.00	0.00	0.00	−0.01
Gly53	0.00	−0.01	0.00	−0.01	0.00	0.00	0.00	0.00
Ser54	0.00	−0.02	0.00	0.01	0.00	0.00	0.00	0.02
Asp55	−0.02	−0.09	−0.02	0.10	−0.01	−0.05	−0.03	−0.02
Arg56	−0.01	−0.05	−0.01	−0.06	−0.01	0.00	−0.03	0.08
Asn57	−0.05	0.14	−0.04	0.06	−0.05	0.09	−0.18	0.16
Ala58	−0.01	0.03	−0.01	0.02	−0.01	0.01	−0.02	−0.04
Ala59	−0.05	0.16	−0.04	0.05	−0.07	0.08	−0.33	0.07
His60	−1.30	−0.15	−0.47	0.09	−2.97	1.20	−3.09	2.57
Met61	−0.40	0.13	−0.21	−0.23	−0.20	0.10	−1.49	0.57
Ser62	−0.01	0.03	−0.01	0.06	−0.01	0.01	−0.05	−0.04
Ser63	0.00	0.01	0.00	0.00	0.00	0.01	−0.01	0.00
Ala64	−0.01	0.04	0.00	0.01	−0.01	0.03	−0.02	0.08
Phe65	−0.24	−0.01	−0.12	0.02	−0.22	0.01	−1.07	−0.28
Leu66	−0.01	0.05	−0.01	0.04	−0.01	0.04	−0.04	0.09
Pro67	0.00	0.01	0.00	−0.01	0.00	0.01	−0.01	0.03
Trp68	−0.04	−0.02	−0.03	−0.01	−0.03	0.00	−0.06	0.04
His69	−0.21	0.46	−0.09	0.25	−0.18	0.26	−0.43	0.74
Arg70	0.00	−0.01	0.00	−0.02	0.00	0.01	−0.01	0.03
Glu71	0.00	−0.02	0.00	−0.03	0.00	−0.01	0.00	−0.01
Tyr72	−0.03	0.01	−0.02	0.00	−0.03	0.03	−0.02	0.02
Leu73	−0.01	−0.03	−0.01	−0.03	−0.01	−0.01	−0.01	−0.02
Leu74	0.00	−0.01	0.00	−0.01	0.00	0.00	0.00	0.00
Arg75	0.00	−0.03	0.00	−0.05	0.00	−0.03	0.00	−0.05
Phe76	0.00	−0.03	0.00	−0.03	0.00	−0.02	0.00	−0.02
Glu77	0.00	−0.02	0.00	−0.02	0.00	−0.01	0.00	−0.01
Arg78	0.00	−0.01	0.00	−0.01	0.00	0.00	0.00	−0.01
Asp79	0.00	−0.02	0.00	−0.01	0.00	0.00	0.00	0.00
Leu80	0.00	−0.01	0.00	−0.02	0.00	−0.01	0.00	−0.01
Gln81	0.00	−0.01	0.00	−0.01	0.00	−0.01	0.00	−0.01
Ser82	0.00	0.00	0.00	0.00	0.00	0.00	0.00	0.00
Ile83	0.00	0.00	0.00	−0.01	0.00	−0.01	0.00	−0.01
Asn84	0.00	0.01	0.00	0.01	0.00	0.00	0.00	0.00
Pro85	0.00	0.00	0.00	0.00	0.00	0.00	0.00	0.00
Glu86	0.00	0.00	0.00	0.00	0.00	0.00	0.00	-0.01
Val87	0.00	0.00	0.00	0.00	0.00	0.00	0.00	0.00
Thr88	0.00	0.00	0.00	0.00	0.00	0.00	0.00	0.00
Leu89	0.00	0.00	0.00	0.00	0.00	-0.01	0.00	-0.01
Pro90	0.00	0.03	0.00	0.03	0.00	0.02	0.00	0.02
Tyr91	0.00	−0.05	0.00	-0.05	0.00	-0.03	0.00	-0.01
Trp92	−0.01	0.04	-0.01	0.03	-0.01	0.01	-0.01	-0.01
Glu93	0.00	-0.02	0.00	-0.02	0.00	-0.01	0.00	0.00
Trp94	-0.02	-0.05	-0.02	-0.05	-0.02	-0.05	-0.01	-0.02
Glu95	0.00	0.00	0.00	0.00	0.00	0.00	0.00	0.00
Thr96	0.00	-0.02	0.00	-0.02	0.00	-0.01	0.00	0.00
Asp97	0.00	-0.02	0.00	-0.02	0.00	0.00	0.00	0.00
Ala98	0.00	-0.02	0.00	-0.02	0.00	0.00	0.00	0.00
Gln99	0.00	-0.03	0.00	-0.02	0.00	0.00	0.00	0.01
Met100	0.00	0.02	0.00	0.01	0.00	0.00	0.00	0.00
Gln101	0.00	0.02	0.00	0.00	0.00	0.00	0.00	-0.01
Asp102	0.00	-0.01	0.00	0.00	0.00	0.00	0.00	0.00

Pro103	0.00	0.00	0.00	0.01	0.00	0.01	0.00	0.01
Ser104	0.00	-0.01	0.00	0.00	0.00	0.00	0.00	0.00
Gln105	0.00	0.00	0.00	0.01	0.00	0.01	0.00	0.01
Ser106	0.00	-0.02	0.00	-0.01	0.00	0.00	0.00	0.01
Gln107	0.00	0.00	0.00	0.00	0.00	0.00	0.00	0.00
Ile108	0.00	-0.01	0.00	-0.01	0.00	0.00	0.00	0.01
Trp109	-0.01	-0.02	-0.01	-0.01	0.00	0.00	0.00	0.00
Ser110	0.00	0.00	0.00	0.00	0.00	0.01	0.00	0.01
Ala111	0.00	0.00	0.00	0.00	0.00	0.00	0.00	0.00
Asp112	0.00	0.01	0.00	0.01	0.00	0.00	0.00	0.00
Phe113	0.00	-0.01	0.00	0.00	0.00	0.00	0.00	0.01
Met114	0.00	0.00	0.00	0.01	0.00	0.03	0.00	0.02
Gly115	0.00	0.02	0.00	0.02	0.00	0.01	0.00	0.01
Gly116	0.00	-0.01	0.00	0.00	0.00	0.01	0.00	0.01
Asn117	0.00	-0.02	0.00	0.00	0.00	0.01	0.00	0.01
Gly118	0.00	0.02	0.00	0.01	0.00	0.00	0.00	0.00
Asn119	0.00	-0.01	0.00	-0.01	0.00	-0.01	0.00	0.00
Pro120	0.00	-0.01	0.00	-0.01	0.00	0.00	0.00	0.00
Ile121	0.00	-0.01	0.00	-0.01	0.00	0.00	0.00	0.00
Lys122	0.00	0.00	0.00	0.00	0.00	0.00	0.00	0.00
Asp123	0.00	-0.04	0.00	-0.02	0.00	0.01	0.00	0.01
Phe124	0.00	-0.01	0.00	-0.01	0.00	0.00	0.00	0.00
Ile125	0.00	0.02	0.00	0.00	0.00	-0.01	0.00	-0.01
Val126	0.00	-0.01	0.00	-0.01	0.00	0.00	0.00	0.00
Asp127	0.00	0.00	0.00	0.00	0.00	-0.01	0.00	-0.01
Thr128	0.00	0.00	0.00	0.00	0.00	0.01	0.00	0.01
Gly129	0.00	-0.01	0.00	-0.01	0.00	0.00	0.00	0.00
Pro130	0.00	-0.01	0.00	-0.01	0.00	0.00	0.00	0.00
Phe131	0.00	-0.01	0.00	-0.01	0.00	0.00	0.00	0.00
Ala132	0.00	0.00	0.00	0.01	0.00	0.01	0.00	0.01
Ala133	0.00	0.01	0.00	0.01	0.00	0.00	0.00	0.00
Gly134	0.00	0.00	0.00	0.00	0.00	0.00	0.00	0.00
Arg135	0.00	0.00	0.00	0.00	0.00	0.00	0.00	0.00
Trp136	0.00	0.00	0.00	-0.01	0.00	-0.01	0.00	-0.01
Thr137	0.00	0.01	0.00	0.02	0.00	0.02	0.00	0.02
Thr138	0.00	0.02	0.00	0.01	0.00	-0.03	0.00	-0.02
Ile139	0.00	-0.04	0.00	-0.02	0.00	0.02	0.00	0.01
Asp140	-0.01	0.04	-0.01	0.04	0.00	0.02	0.00	0.02
Glu141	-0.02	0.04	-0.01	0.05	-0.01	0.01	-0.01	0.02
Gln142	0.00	0.04	0.00	0.05	0.00	0.01	0.00	0.00
Gly143	0.00	0.03	0.00	0.02	0.00	-0.01	0.00	-0.01
Asn144	0.00	-0.01	0.00	0.01	0.00	0.02	0.00	0.01
Pro145	0.00	-0.01	0.00	-0.01	0.00	-0.01	0.00	0.00
Ser146	0.00	0.00	0.00	-0.02	0.00	0.00	0.00	-0.01
Gly147	0.00	0.00	0.00	-0.01	0.00	-0.01	0.00	-0.01
Gly148	0.00	-0.03	0.00	-0.03	0.00	-0.01	0.00	0.00
Leu149	-0.01	0.02	-0.01	0.00	-0.01	-0.02	0.00	-0.01
Lys150	-0.01	0.01	-0.01	0.03	-0.01	0.03	0.00	0.01
Arg151	-0.01	-0.03	-0.01	0.01	-0.01	0.00	0.00	0.01
Asn152	-0.01	0.03	-0.01	0.02	0.00	-0.01	0.00	-0.01
Phe153	-0.01	-0.03	-0.01	-0.02	-0.01	0.00	0.00	0.00
Gly154	0.00	-0.02	0.00	0.00	0.00	0.01	0.00	0.01
Ala155	-0.01	-0.01	-0.01	-0.01	0.00	0.00	0.00	0.00
Thr156	-0.03	0.09	-0.03	0.07	-0.01	-0.01	-0.01	-0.02
Lys157	-0.01	0.03	-0.01	0.04	0.00	0.00	0.00	-0.01
Glu158	-0.08	0.03	-0.07	0.13	-0.01	0.00	-0.02	0.01
Ala159	-0.04	-0.01	-0.04	-0.01	-0.01	0.00	-0.01	0.01
Pro160	-0.01	-0.02	-0.01	0.01	0.00	0.01	0.00	0.01

Thr161	-0.01	0.06	-0.01	0.01	0.00	-0.03	0.00	-0.03
Leu162	-0.01	-0.03	-0.01	-0.04	-0.01	-0.01	0.00	0.00
Pro163	-0.01	-0.03	-0.01	0.00	-0.01	0.02	-0.01	0.03
Thr164	0.00	0.04	0.00	0.04	0.00	0.00	0.00	-0.02
Arg165	0.00	0.01	0.00	0.00	0.00	-0.01	0.00	-0.01
Asp166	0.00	0.01	0.00	0.01	0.00	0.00	0.00	-0.01
Asp167	0.00	0.00	0.00	0.01	0.00	0.00	0.00	0.00
Val168	0.00	0.00	0.00	0.00	0.00	0.00	0.00	-0.01
Leu169	0.00	0.00	0.00	0.01	0.00	0.00	0.00	0.00
Asn170	0.00	-0.04	0.00	0.00	0.00	0.01	0.00	0.02
Ala171	0.00	-0.02	0.00	-0.01	0.00	0.00	0.00	0.00
Leu172	0.00	-0.01	0.00	0.00	0.00	0.00	0.00	0.00
Lys173	0.00	-0.01	0.00	-0.01	0.00	0.00	0.00	0.01
Ile174	0.00	0.00	0.00	0.00	0.00	0.01	0.00	0.01
Thr175	0.00	0.00	0.00	0.00	0.00	0.00	0.00	0.01
Gln176	0.00	0.01	0.00	0.02	0.00	0.00	0.00	-0.02
Tyr177	0.00	0.04	0.00	0.05	0.00	0.00	-0.01	-0.08
Asp178	0.00	0.01	0.00	0.01	0.00	0.01	0.00	0.01
Thr179	0.00	0.00	0.00	0.01	0.00	-0.01	0.00	-0.02
Pro180	0.00	0.01	0.00	0.00	0.00	0.00	0.00	-0.01
Pro181	0.00	-0.01	0.00	0.01	0.00	0.00	0.00	0.00
Trp182	0.00	-0.02	0.00	0.01	0.00	0.00	-0.01	0.01
Asp183	-0.01	0.04	-0.01	-0.08	-0.01	0.02	-0.02	0.06
Met184	-0.09	-0.06	-0.07	-0.01	-0.02	-0.02	-0.07	0.04
Thr185	-0.01	0.02	-0.01	0.02	0.00	0.00	-0.01	-0.01
Ser186	0.00	0.01	0.00	0.02	0.00	-0.02	0.00	-0.04
Gln187	0.00	0.01	0.00	0.00	0.00	0.01	0.00	0.02
Asn188	0.00	0.00	0.00	0.00	0.00	0.01	0.00	0.02
Ser189	0.00	0.03	0.00	0.01	0.00	-0.01	0.00	0.00
Phe190	-0.01	0.04	0.00	0.03	0.00	-0.01	-0.01	-0.06
Arg191	-0.02	0.08	-0.02	0.14	-0.02	-0.02	-0.06	-0.29
Asn192	-0.03	0.03	-0.02	0.17	-0.01	-0.06	-0.04	-0.31
Gln193	-0.01	0.11	-0.01	0.05	-0.01	-0.04	-0.02	-0.11
Leu194	-0.03	0.09	-0.03	0.04	-0.03	0.00	-0.04	-0.01
Glu195	-0.33	0.05	-0.29	0.31	-0.32	-0.08	1.76	-12.45
Gly196	-0.18	0.26	-0.10	-0.04	-0.05	-0.06	-0.10	-0.10
Phe197	-1.23	0.13	-1.09	-0.08	-0.22	-0.04	-1.22	-0.04
Ile198	-0.02	0.04	-0.01	0.01	-0.01	-0.01	-0.01	-0.02
Asn199	-0.02	0.02	-0.01	0.00	0.00	0.00	-0.01	0.00
Gly200	-0.11	-0.32	-0.05	-0.02	-0.01	0.05	-0.02	0.10
Pro201	-0.83	-0.37	-0.47	0.01	-0.06	0.06	-0.09	0.10
Gln202	-0.12	0.20	-0.08	0.07	-0.04	-0.07	-0.04	-0.11
Leu203	-0.06	0.09	-0.05	-0.01	-0.05	-0.06	-0.04	-0.03
His204	-1.73	1.92	-1.36	0.39	-1.81	1.60	-0.56	1.21
Asn205	-3.79	-1.40	-3.93	0.65	-1.17	-0.40	-1.61	-1.60
Arg206	-0.32	-0.13	-0.24	-0.05	-0.12	-0.05	-0.09	0.02
Val207	-0.17	-0.15	-0.16	-0.14	-0.14	0.00	-0.10	0.10
His208	-4.42	2.44	-3.83	1.33	0.67	1.45	-2.58	1.03
Arg209	-2.24	-0.16	-1.59	1.59	-0.38	0.16	-0.13	0.22
Trp210	-0.06	-0.10	-0.07	-0.09	-0.03	0.00	-0.02	0.02
Val211	-0.05	-0.03	-0.06	-0.05	-0.04	0.00	-0.01	0.01
Gly212	-0.05	0.04	-0.05	0.10	-0.03	0.10	-0.01	0.03
Gly213	-0.04	-0.18	-0.04	-0.15	-0.03	-0.05	-0.01	-0.02
Gln214	-0.18	-0.50	-0.22	-0.36	-0.15	0.07	-0.03	-0.02
Met215	-1.21	-0.03	-1.32	0.25	-1.25	0.14	-0.16	0.02
Gly216	-0.81	-0.50	-1.21	-0.51	-0.97	-0.41	-0.27	-0.13
Val217	-0.79	-0.77	-0.87	-0.49	-1.62	0.81	-0.32	0.07
Val218	-3.33	-0.28	-3.58	-1.81	-1.54	0.34	-1.78	0.27

Pro219	-0.24	0.12	-0.54	-0.05	-0.21	0.06	-0.09	0.04
Thr220	-0.12	0.06	-0.14	-0.04	-0.10	0.03	-0.03	0.00
Ala221	-0.70	0.56	-0.87	0.23	-0.64	-0.40	-0.12	-0.10
Pro222	-0.16	0.05	-0.15	0.00	-0.15	-0.05	-0.04	-0.06
Asn223	-0.02	0.21	-0.02	0.11	-0.02	-0.09	-0.01	-0.03
Asp224	-0.03	0.07	-0.03	0.05	-0.03	0.04	-0.01	0.03
Pro225	-0.01	0.05	-0.01	0.04	-0.01	0.02	0.00	0.02
Val226	-0.02	0.07	-0.02	0.05	-0.02	0.01	-0.01	0.01
Phe227	-0.65	0.13	-0.53	0.05	-0.74	0.00	-0.20	-0.01
Phe228	-0.02	0.11	-0.02	0.09	-0.02	0.06	-0.01	0.05
Leu229	-0.01	0.02	-0.01	0.01	-0.01	-0.01	0.00	-0.01
His230	-0.17	-0.27	-0.18	0.05	-0.23	-0.01	-0.09	-0.07
His231	-0.28	0.89	-0.19	0.48	-0.51	0.65	-0.32	0.42
Ala232	0.00	0.00	0.00	0.01	-0.01	-0.01	0.00	-0.01
Asn233	-0.01	-0.12	-0.01	-0.08	-0.01	-0.04	-0.01	-0.02
Val234	-0.03	-0.09	-0.02	-0.07	-0.04	-0.06	-0.03	-0.05
Asp235	0.00	-0.03	0.00	-0.03	0.00	-0.02	-0.01	-0.02
Arg236	0.00	-0.07	0.00	-0.06	0.00	-0.03	0.00	-0.01
Ile237	0.00	-0.04	0.00	-0.03	0.00	-0.01	0.00	0.00
Trp238	0.00	-0.02	0.00	-0.01	0.00	-0.02	-0.01	-0.03
Ala239	0.00	-0.02	0.00	-0.01	0.00	-0.01	0.00	-0.01
Val240	0.00	-0.02	0.00	-0.02	0.00	0.00	0.00	0.00
Trp241	0.00	-0.02	0.00	-0.02	0.00	0.00	0.00	0.01
Gln242	0.00	0.00	0.00	-0.01	0.00	0.00	0.00	0.01
Ile243	0.00	-0.01	0.00	-0.01	0.00	0.00	0.00	0.00
Ile244	0.00	-0.01	0.00	-0.01	0.00	0.00	0.00	0.01
His245	0.00	0.01	0.00	0.01	0.00	0.00	0.00	-0.01
Arg246	0.00	0.00	0.00	0.00	0.00	0.00	0.00	-0.01
Asn247	0.00	0.00	0.00	0.00	0.00	0.00	0.00	0.00
Gln248	0.00	0.00	0.00	0.01	0.00	0.00	0.00	0.00
Asn249	0.00	0.01	0.00	0.02	0.00	0.00	0.00	-0.01
Tyr250	0.00	-0.02	0.00	-0.01	0.00	-0.01	0.00	-0.02
Gln251	0.00	0.00	0.00	0.01	0.00	0.00	0.00	-0.01
Pro252	0.00	0.01	0.00	0.01	0.00	0.00	0.00	0.01
Met253	0.00	0.01	0.00	0.01	0.00	0.00	0.00	0.00
Lys254	0.00	0.01	0.00	0.00	0.00	0.00	0.00	-0.01
Asn255	0.00	0.00	0.00	0.00	0.00	0.00	0.00	-0.01
Gly256	0.00	0.00	0.00	-0.02	0.00	0.00	0.00	0.02
Pro257	0.00	0.01	0.00	0.01	0.00	0.01	0.00	0.00
Phe258	0.00	-0.01	0.00	0.02	0.00	-0.01	0.00	0.00
Gly259	0.00	0.01	0.00	0.02	0.00	0.00	0.00	0.01
Gln260	0.00	0.05	0.00	0.03	0.00	0.02	-0.01	-0.04
Asn261	0.00	0.01	0.00	0.03	0.00	0.00	0.00	-0.01
Phe262	0.00	0.01	0.00	0.01	0.00	0.01	0.00	0.01
Arg263	0.00	0.00	0.00	0.00	0.00	0.00	0.00	0.00
Asp264	0.00	0.00	0.00	0.01	0.00	0.00	0.00	0.00
Pro265	0.00	-0.01	0.00	0.00	0.00	-0.01	0.00	-0.01
Met266	0.00	0.02	0.00	0.01	0.00	0.01	0.00	0.00
Tyr267	0.00	0.03	0.00	0.04	0.00	0.01	-0.01	0.03
Pro268	0.00	0.00	0.00	-0.02	0.00	0.00	0.00	-0.02
Trp269	0.00	-0.02	0.00	-0.01	0.00	-0.02	-0.01	-0.02
Asn270	0.00	-0.01	0.00	0.00	0.00	-0.01	0.00	0.00
Thr271	0.00	0.00	0.00	0.00	0.00	0.00	0.00	0.00
Thr272	0.00	0.02	0.00	0.01	0.00	0.01	0.00	0.01
Pro273	0.00	0.01	0.00	0.00	0.00	0.01	0.00	0.01
Glu274	0.00	0.01	0.00	0.00	0.00	0.00	0.00	0.00
Asp275	0.00	0.00	0.00	0.00	0.00	0.00	0.00	0.00
Val276	0.00	0.01	0.00	0.01	0.00	0.00	0.00	0.00

Met277	0.00	0.02	0.00	0.02	0.00	0.00	0.00	-0.01
Asn278	0.00	0.01	0.00	0.01	0.00	0.01	0.00	0.01
His279	0.00	-0.01	0.00	-0.01	0.00	0.00	0.00	0.01
Arg280	0.00	0.00	0.00	0.00	0.00	0.00	0.00	0.00
Lys281	0.00	-0.01	0.00	-0.01	0.00	0.00	0.00	0.00
Leu282	0.00	-0.01	0.00	-0.01	0.00	0.00	0.00	0.00
Gly283	0.00	0.00	0.00	0.00	0.00	0.00	0.00	0.00
Tyr284	0.00	-0.01	0.00	-0.01	0.00	-0.01	0.00	-0.01
Val285	0.00	0.00	0.00	0.00	0.00	0.00	0.00	0.01
Tyr286	0.00	0.00	0.00	0.00	0.00	0.00	0.00	-0.01
Asp287	0.00	-0.01	0.00	-0.01	0.00	0.00	0.00	0.00
Ile288	0.00	-0.01	0.00	-0.01	0.00	-0.01	0.00	0.00
Glu289	0.00	-0.02	0.00	-0.01	0.00	-0.01	0.00	0.00
Leu290	0.00	0.00	0.00	0.00	0.00	0.00	0.00	0.00
CuB291	-0.38	-4.16	-0.34	-7.39	-0.24	-1.98	-0.62	-11.88
CuA292	-2.63	-17.63	-0.87	-10.80	-5.65	-15.04	-0.45	-5.70

Table S3. Individual polar and non-polar residual contributions of KA analogs systems. The values are reported in the kcal/mol.

Residue	KA1		KA2		KA3		KA4		KA5	
	V_{vdw}	V_{ele}	V_{vdw}	V_{ele}	V_{vdw}	V_{ele}	V_{vdw}	V_{ele}	V_{vdw}	V_{ele}
Tyr5	0.00	0.00	0.00	0.00	0.00	0.00	0.00	0.00	0.00	0.00
Arg6	0.00	-0.01	0.00	-0.01	0.00	-0.01	0.00	0.00	0.00	0.00
Val7	0.00	0.00	0.00	0.00	0.00	0.01	0.00	0.00	0.00	0.00
Arg8	0.00	-0.02	0.00	-0.02	0.00	-0.02	0.00	-0.01	0.00	0.00
Lys9	0.00	0.01	0.00	0.01	0.00	0.02	0.00	0.01	0.00	0.00
Asn10	0.00	-0.01	0.00	0.00	0.00	0.01	0.00	0.00	0.00	-0.01
Val11	0.00	-0.01	0.00	-0.01	0.00	-0.01	0.00	-0.01	0.00	0.00
Leu12	0.00	-0.01	0.00	0.00	0.00	0.00	0.00	0.00	0.00	0.00
His13	0.00	-0.01	0.00	-0.01	0.00	-0.02	0.00	0.00	0.00	0.00
Leu14	0.00	0.00	0.00	0.00	0.00	-0.01	0.00	0.00	0.00	0.00
Thr15	0.00	0.01	0.00	0.01	0.00	0.02	0.00	0.01	0.00	0.00
Asp16	0.00	0.00	0.00	0.00	0.00	0.00	0.00	0.00	0.00	0.00
Thr17	0.00	0.00	0.00	0.00	0.00	0.01	0.00	0.00	0.00	0.00
Glu18	0.00	0.00	0.00	0.00	0.00	0.01	0.00	0.00	0.00	0.00
Lys19	0.00	0.00	0.00	0.00	0.00	0.01	0.00	0.00	0.00	0.00
Arg20	0.00	0.00	0.00	0.00	0.00	0.01	0.00	0.00	0.00	0.00
Asp21	0.00	0.00	0.00	0.00	0.00	0.00	0.00	0.00	0.00	0.00
Phe22	0.00	0.00	0.00	0.00	0.00	0.01	0.00	0.00	0.00	0.00
Val23	0.00	0.00	0.00	0.01	0.00	0.01	0.00	0.00	0.00	0.00
Arg24	0.00	0.00	0.00	0.00	0.00	0.01	0.00	0.00	0.00	-0.01
Thr25	0.00	0.00	0.00	0.00	0.00	0.00	0.00	0.00	0.00	0.00
Val26	0.00	-0.01	0.00	0.00	0.00	0.00	0.00	0.00	0.00	0.00
Leu27	0.00	0.00	0.00	0.00	0.00	0.01	0.00	0.00	0.00	0.00
Ile28	0.00	-0.01	0.00	0.00	0.00	0.00	0.00	0.00	0.00	0.00
Leu29	0.00	-0.01	0.00	-0.01	0.00	-0.01	0.00	0.00	0.00	0.00
Lys30	0.00	-0.01	0.00	-0.02	0.00	-0.02	0.00	-0.01	0.00	0.01
Glu31	0.00	-0.01	0.00	-0.01	0.00	0.00	0.00	0.00	0.00	0.00
Lys32	0.00	0.00	0.00	-0.01	0.00	-0.01	0.00	0.00	0.00	0.00
Gly33	0.00	0.00	0.00	0.00	0.00	0.00	0.00	0.00	0.00	0.00
Ile34	0.00	0.00	0.00	0.01	0.00	0.02	0.00	0.00	0.00	0.00
Tyr35	0.00	0.02	0.00	0.01	-0.01	0.01	-0.01	0.00	-0.01	0.01
Asp36	0.00	0.00	0.00	0.01	0.00	0.03	0.00	0.00	0.00	-0.01
Arg37	0.00	-0.02	0.00	-0.01	0.00	0.01	0.00	-0.01	0.00	-0.01
Tyr38	-0.01	0.01	-0.01	0.00	-0.01	0.03	-0.01	-0.01	-0.01	0.01
Ile39	-0.02	0.01	-0.02	0.02	-0.02	0.08	-0.02	0.01	-0.02	-0.02
Ala40	0.00	-0.01	0.00	0.00	-0.01	0.04	-0.01	-0.01	-0.01	-0.02
Trp41	-0.01	-0.03	-0.01	-0.06	-0.01	-0.03	-0.01	-0.05	-0.01	0.02

His42	-0.69	-0.07	-0.81	-0.67	-1.18	-0.22	-1.15	-0.37	-1.47	1.04
Gly43	-0.02	-0.01	-0.02	-0.08	-0.02	-0.02	-0.02	-0.07	-0.03	0.03
Ala44	-0.01	-0.03	-0.01	-0.05	-0.01	-0.04	-0.01	-0.04	-0.01	0.01
Ala45	-0.01	-0.04	-0.02	-0.06	-0.02	-0.11	-0.02	-0.03	-0.02	0.03
Gly46	-0.02	-0.02	-0.03	-0.05	-0.04	-0.12	-0.04	0.00	-0.04	0.06
Lys47	-0.01	0.00	-0.01	-0.02	-0.01	-0.04	-0.01	-0.01	-0.01	0.01
Phe48	0.00	-0.01	-0.01	-0.03	-0.01	-0.02	-0.01	-0.02	-0.01	0.02
His49	-0.01	0.05	-0.01	0.08	-0.01	0.08	-0.01	0.03	-0.01	-0.04
Thr50	0.00	-0.01	0.00	-0.04	0.00	-0.02	0.00	-0.03	0.00	0.02
Pro51	0.00	-0.02	0.00	-0.03	0.00	-0.02	0.00	-0.01	0.00	0.01
Pro52	0.00	0.01	0.00	0.01	0.00	0.00	0.00	0.01	0.00	0.00
Gly53	0.00	0.00	0.00	0.00	0.00	-0.01	0.00	0.00	0.00	0.00
Ser54	0.00	-0.02	0.00	-0.03	0.00	0.00	0.00	-0.04	0.00	0.01
Asp55	-0.01	-0.14	-0.01	-0.11	-0.02	-0.08	-0.01	-0.03	-0.01	0.04
Arg56	-0.01	0.00	-0.01	0.05	-0.01	0.04	-0.01	0.01	-0.01	-0.02
Asn57	-0.05	0.16	-0.05	0.00	-0.07	0.07	-0.05	-0.04	-0.05	0.03
Ala58	-0.01	0.01	-0.01	0.00	-0.01	-0.02	-0.01	0.01	-0.01	0.02
Ala59	-0.09	0.16	-0.07	0.03	-0.10	0.04	-0.08	-0.01	-0.12	0.19
His60	-1.25	1.11	-0.54	-0.65	-0.02	0.16	-0.43	-1.39	-1.88	0.06
Met61	-0.23	0.28	-0.18	0.33	-0.33	0.28	-0.20	0.06	-0.17	-0.11
Ser62	-0.01	0.00	-0.01	-0.03	-0.02	0.00	-0.01	-0.03	-0.01	0.03
Ser63	0.00	0.01	0.00	0.01	0.00	0.01	0.00	0.00	0.00	0.01
Ala64	-0.01	0.04	-0.01	0.04	-0.01	0.05	-0.01	0.02	-0.01	0.01
Phe65	-0.15	0.04	-0.27	0.04	-0.37	0.07	-0.28	0.08	-0.25	-0.01
Leu66	-0.01	0.03	-0.01	0.02	-0.02	0.06	-0.01	-0.01	-0.02	0.05
Pro67	0.00	0.01	0.00	0.03	-0.01	0.03	0.00	0.02	-0.01	-0.01
Trp68	-0.03	0.00	-0.04	0.01	-0.05	0.01	-0.05	0.00	-0.06	-0.03
His69	-0.13	0.21	-0.22	0.41	-0.22	0.58	-0.25	0.21	-0.43	0.42
Arg70	0.00	0.01	0.00	0.04	0.00	0.03	0.00	0.02	-0.01	-0.02
Glu71	0.00	-0.01	0.00	0.01	0.00	-0.01	0.00	0.01	0.00	-0.03
Tyr72	-0.02	0.03	-0.03	0.10	-0.03	0.10	-0.03	0.09	-0.05	-0.08
Leu73	-0.01	-0.01	-0.01	0.00	-0.01	-0.01	-0.01	0.00	-0.01	-0.02
Leu74	0.00	0.00	0.00	0.01	0.00	0.00	0.00	0.01	0.00	-0.02
Arg75	0.00	-0.02	0.00	-0.02	0.00	-0.06	0.00	0.00	0.00	-0.01
Phe76	0.00	-0.02	0.00	-0.01	0.00	-0.03	0.00	0.00	0.00	-0.01
Glu77	0.00	-0.01	0.00	0.00	0.00	-0.01	0.00	0.00	0.00	-0.01
Arg78	0.00	-0.01	0.00	0.00	0.00	-0.01	0.00	0.00	0.00	-0.01
Asp79	0.00	-0.01	0.00	0.00	0.00	0.00	0.00	0.00	0.00	0.00
Leu80	0.00	-0.01	0.00	-0.01	0.00	-0.02	0.00	-0.01	0.00	0.00
Gln81	0.00	-0.01	0.00	-0.01	0.00	-0.02	0.00	-0.01	0.00	0.00
Ser82	0.00	0.00	0.00	0.00	0.00	0.00	0.00	0.00	0.00	0.00
Ile83	0.00	0.00	0.00	-0.01	0.00	-0.01	0.00	0.00	0.00	0.00
Asn84	0.00	0.00	0.00	0.00	0.00	0.00	0.00	0.00	0.00	0.00
Pro85	0.00	0.00	0.00	0.00	0.00	0.00	0.00	0.00	0.00	0.00
Glu86	0.00	0.00	0.00	-0.01	0.00	-0.01	0.00	0.00	0.00	0.00
Val87	0.00	0.00	0.00	0.00	0.00	0.00	0.00	0.00	0.00	0.00
Thr88	0.00	0.00	0.00	0.01	0.00	0.01	0.00	0.00	0.00	-0.01
Leu89	0.00	0.00	0.00	-0.01	0.00	-0.02	0.00	0.00	0.00	0.01
Pro90	0.00	0.02	0.00	0.02	0.00	0.04	0.00	0.01	0.00	0.00
Tyr91	0.00	-0.04	0.00	-0.02	0.00	-0.03	0.00	-0.01	0.00	-0.01
Trp92	-0.01	0.02	-0.01	0.00	-0.01	0.00	-0.01	0.01	-0.01	0.01
Glu93	0.00	-0.02	0.00	-0.01	0.00	-0.01	0.00	-0.01	0.00	0.01
Trp94	-0.02	-0.03	-0.02	-0.03	-0.02	-0.06	-0.02	0.00	-0.02	-0.01
Glu95	0.00	-0.01	0.00	-0.01	0.00	-0.01	0.00	-0.01	0.00	0.01
Thr96	0.00	-0.01	0.00	-0.01	0.00	-0.01	0.00	-0.01	0.00	0.00
Asp97	0.00	-0.01	0.00	0.00	0.00	-0.01	0.00	-0.01	0.00	0.00
Ala98	0.00	-0.01	0.00	0.00	0.00	0.00	0.00	-0.01	0.00	0.00
Gln99	0.00	-0.02	0.00	-0.01	0.00	0.00	0.00	-0.01	0.00	0.00

Met100	0.00	0.01	0.00	0.01	0.00	0.00	0.00	0.01	0.00	0.00
Gln101	0.00	0.01	0.00	0.00	0.00	-0.01	0.00	0.01	0.00	0.00
Asp102	0.00	-0.01	0.00	0.00	0.00	0.01	0.00	0.00	0.00	0.01
Pro103	0.00	0.00	0.00	0.00	0.00	0.02	0.00	0.00	0.00	0.00
Ser104	0.00	-0.01	0.00	0.00	0.00	0.00	0.00	0.00	0.00	0.00
Gln105	0.00	0.00	0.00	0.00	0.00	0.01	0.00	0.00	0.00	0.00
Ser106	0.00	-0.01	0.00	0.00	0.00	0.00	0.00	-0.01	0.00	0.00
Gln107	0.00	0.00	0.00	0.00	0.00	0.01	0.00	0.00	0.00	0.00
Ile108	0.00	-0.01	0.00	0.00	0.00	0.01	0.00	0.00	0.00	0.00
Trp109	0.00	-0.01	0.00	-0.01	0.00	0.00	-0.01	-0.01	0.00	0.01
Ser110	0.00	0.00	0.00	0.01	0.00	0.02	0.00	0.00	0.00	-0.01
Ala111	0.00	0.00	0.00	0.00	0.00	0.01	0.00	0.00	0.00	0.00
Asp112	0.00	0.00	0.00	0.00	0.00	0.00	0.00	0.00	0.00	0.01
Phe113	0.00	0.00	0.00	0.00	0.00	0.01	0.00	0.00	0.00	0.00
Met114	0.00	0.01	0.00	0.03	0.00	0.06	0.00	0.01	0.00	-0.03
Gly115	0.00	0.02	0.00	0.02	0.00	0.02	0.00	0.01	0.00	-0.01
Gly116	0.00	0.00	0.00	0.01	0.00	0.03	0.00	0.00	0.00	-0.02
Asn117	0.00	-0.01	0.00	0.01	0.00	0.03	0.00	-0.01	0.00	-0.02
Gly118	0.00	0.01	0.00	0.00	0.00	-0.01	0.00	0.01	0.00	0.01
Asn119	0.00	0.00	0.00	0.00	0.00	-0.01	0.00	-0.01	0.00	0.00
Pro120	0.00	-0.01	0.00	0.00	0.00	0.00	0.00	0.00	0.00	0.00
Ile121	0.00	0.00	0.00	0.00	0.00	0.00	0.00	0.00	0.00	0.00
Lys122	0.00	0.00	0.00	0.00	0.00	0.00	0.00	0.00	0.00	0.00
Asp123	0.00	-0.02	0.00	0.00	0.00	0.02	0.00	-0.02	0.00	-0.02
Phe124	0.00	0.00	0.00	0.01	0.00	0.01	0.00	-0.01	0.00	-0.02
Ile125	0.00	0.01	0.00	0.00	0.00	-0.02	0.00	0.00	0.00	0.01
Val126	0.00	-0.01	0.00	0.00	0.00	0.00	0.00	0.00	0.00	0.01
Asp127	0.00	0.00	0.00	-0.01	0.00	-0.02	0.00	0.00	0.00	0.01
Thr128	0.00	0.00	0.00	0.01	0.00	0.02	0.00	0.00	0.00	-0.02
Gly129	0.00	-0.01	0.00	0.00	0.00	0.00	0.00	-0.01	0.00	0.00
Pro130	0.00	-0.01	0.00	0.00	0.00	0.00	0.00	0.00	0.00	0.00
Phe131	0.00	0.00	0.00	0.00	0.00	0.01	0.00	0.00	0.00	-0.01
Ala132	0.00	0.00	0.00	0.01	0.00	0.02	0.00	0.01	0.00	0.00
Ala133	0.00	0.01	0.00	0.00	0.00	0.00	0.00	0.00	0.00	0.00
Gly134	0.00	0.00	0.00	0.00	0.00	-0.01	0.00	0.00	0.00	0.01
Arg135	0.00	0.00	0.00	0.00	0.00	0.00	0.00	0.00	0.00	0.00
Trp136	0.00	0.00	0.00	-0.02	0.00	-0.03	0.00	-0.01	0.00	0.01
Thr137	0.00	0.01	0.00	0.03	0.00	0.05	0.00	0.02	0.00	-0.02
Thr138	0.00	0.01	0.00	-0.04	0.00	-0.07	0.00	-0.02	0.00	0.05
Ile139	0.00	-0.02	0.00	0.02	0.00	0.04	0.00	0.01	-0.01	-0.04
Asp140	0.00	0.04	0.00	0.02	0.00	0.03	0.00	0.03	-0.01	0.00
Glu141	-0.01	0.02	-0.01	-0.01	-0.01	0.02	-0.01	0.01	-0.02	0.01
Gln142	0.00	0.03	0.00	0.00	0.00	0.01	0.00	0.02	0.00	0.01
Gly143	0.00	0.01	0.00	-0.01	0.00	-0.02	0.00	0.00	0.00	0.02
Asn144	0.00	0.00	0.00	0.02	0.00	0.04	0.00	0.01	0.00	-0.02
Pro145	0.00	-0.01	0.00	-0.01	0.00	-0.02	0.00	-0.01	0.00	0.01
Ser146	0.00	-0.01	0.00	0.00	0.00	-0.01	0.00	-0.01	0.00	-0.01
Gly147	0.00	0.00	0.00	-0.01	0.00	-0.01	0.00	-0.01	0.00	0.00
Gly148	0.00	-0.02	0.00	-0.01	0.00	-0.01	0.00	-0.02	0.00	-0.01
Leu149	-0.01	0.01	-0.01	-0.02	-0.01	-0.04	-0.01	0.00	-0.01	0.03
Lys150	-0.01	0.00	-0.01	0.02	-0.01	0.05	-0.01	0.02	-0.01	-0.03
Arg151	-0.01	-0.01	-0.01	-0.01	-0.01	0.00	-0.01	0.02	-0.01	0.03
Asn152	-0.01	0.02	-0.01	0.00	-0.01	-0.01	-0.01	0.02	-0.01	0.00
Phe153	-0.01	-0.02	-0.01	-0.01	-0.01	0.00	-0.01	-0.01	-0.01	0.02
Gly154	0.00	-0.02	0.00	-0.01	0.00	0.02	0.00	0.01	0.00	0.03
Ala155	0.00	-0.01	0.00	0.00	0.00	0.00	0.00	0.00	0.00	0.02
Thr156	-0.01	0.05	-0.01	0.01	-0.01	-0.02	-0.02	0.06	-0.01	-0.01
Lys157	0.00	0.01	0.00	0.00	0.00	0.01	0.00	0.01	0.00	0.00

Glu158	-0.02	-0.01	-0.02	-0.02	-0.02	0.03	-0.03	0.05	-0.02	0.04
Ala159	-0.02	-0.01	-0.02	0.00	-0.02	0.01	-0.03	-0.02	-0.01	-0.01
Pro160	0.00	-0.01	0.00	0.00	0.00	0.02	-0.01	-0.02	0.00	-0.01
Thr161	0.00	0.04	0.00	0.01	0.00	-0.05	-0.01	0.04	0.00	-0.01
Leu162	-0.01	-0.03	-0.01	-0.01	-0.01	-0.02	-0.01	-0.01	-0.01	0.01
Pro163	-0.01	-0.01	-0.01	0.00	-0.01	0.03	-0.01	-0.02	-0.01	0.00
Thr164	0.00	0.02	0.00	0.00	0.00	0.00	0.00	0.01	0.00	0.01
Arg165	0.00	0.00	0.00	-0.01	0.00	-0.01	0.00	0.00	0.00	0.00
Asp166	0.00	0.00	0.00	-0.01	0.00	-0.01	0.00	0.00	0.00	0.01
Asp167	0.00	0.00	0.00	-0.01	0.00	0.00	0.00	0.00	0.00	0.00
Val168	0.00	-0.01	0.00	-0.01	0.00	-0.01	0.00	0.00	0.00	0.01
Leu169	0.00	0.00	0.00	0.00	0.00	0.00	0.00	0.00	0.00	0.01
Asn170	0.00	-0.02	0.00	-0.01	0.00	0.02	0.00	-0.03	0.00	0.01
Ala171	0.00	-0.01	0.00	-0.01	0.00	0.00	0.00	-0.01	0.00	0.00
Leu172	0.00	0.00	0.00	0.00	0.00	0.00	0.00	0.00	0.00	0.00
Lys173	0.00	-0.01	0.00	0.00	0.00	0.01	0.00	-0.01	0.00	0.00
Ile174	0.00	0.00	0.00	0.01	0.00	0.01	0.00	0.00	0.00	0.00
Thr175	0.00	0.00	0.00	0.00	0.00	0.01	0.00	0.00	0.00	0.00
Gln176	0.00	0.00	0.00	-0.01	0.00	-0.01	0.00	0.00	0.00	0.01
Tyr177	0.00	0.01	0.00	-0.01	0.00	-0.03	0.00	0.00	0.00	0.02
Asp178	0.00	0.01	0.00	0.01	0.00	0.02	0.00	-0.01	0.00	0.00
Thr179	0.00	-0.01	0.00	-0.01	0.00	-0.02	0.00	0.00	0.00	0.01
Pro180	0.00	0.01	0.00	0.01	0.00	-0.01	0.00	0.01	0.00	0.00
Pro181	0.00	-0.01	0.00	-0.01	0.00	0.00	0.00	-0.01	0.00	0.01
Trp182	0.00	-0.02	0.00	-0.03	0.00	0.00	0.00	-0.03	0.00	0.01
Asp183	-0.01	0.05	-0.01	0.07	-0.01	0.05	-0.01	0.02	0.00	-0.03
Met184	-0.03	0.00	-0.02	0.02	-0.03	-0.06	-0.02	0.05	-0.02	0.01
Thr185	0.00	0.01	0.00	0.00	0.00	0.01	0.00	0.00	0.00	0.00
Ser186	0.00	-0.01	0.00	-0.02	0.00	-0.04	0.00	0.01	0.00	0.01
Gln187	0.00	0.01	0.00	0.01	0.00	0.03	0.00	-0.01	0.00	0.00
Asn188	0.00	0.00	0.00	0.01	0.00	0.02	0.00	-0.01	0.00	0.00
Ser189	0.00	0.02	0.00	0.01	0.00	0.00	0.00	0.01	0.00	0.00
Phe190	0.00	0.02	0.00	-0.01	0.00	-0.02	0.00	0.02	0.00	0.01
Arg191	-0.02	0.01	-0.02	-0.08	-0.02	-0.05	-0.02	-0.02	-0.02	0.04
Asn192	-0.02	-0.05	-0.01	-0.11	-0.02	-0.15	-0.02	0.04	-0.01	0.04
Gln193	-0.01	0.04	-0.01	-0.01	-0.01	-0.09	-0.01	0.10	-0.01	-0.01
Leu194	-0.02	0.07	-0.02	0.03	-0.03	-0.02	-0.02	0.05	-0.02	-0.02
Glu195	-0.37	-0.01	-0.26	-0.42	-0.50	-0.22	-0.25	-0.01	-0.22	0.00
Gly196	-0.08	0.07	-0.05	0.02	-0.07	-0.16	-0.07	0.19	-0.04	-0.01
Phe197	-0.38	0.04	-0.20	0.05	-0.36	-0.09	-0.26	0.19	-0.16	-0.04
Ile198	-0.01	0.02	-0.01	0.01	-0.01	-0.03	-0.01	0.04	-0.01	0.00
Asn199	-0.01	0.02	-0.01	0.01	-0.01	0.00	-0.01	0.01	0.00	-0.01
Gly200	-0.02	-0.07	-0.01	-0.02	-0.02	0.14	-0.02	-0.19	-0.01	0.00
Pro201	-0.19	-0.10	-0.10	-0.03	-0.11	0.16	-0.20	-0.23	-0.07	0.00
Gln202	-0.06	0.14	-0.04	0.02	-0.05	-0.11	-0.06	0.19	-0.03	-0.05
Leu203	-0.05	0.09	-0.04	0.05	-0.05	-0.08	-0.05	0.09	-0.04	-0.08
His204	-1.27	0.70	-1.60	1.52	-0.44	1.33	-1.22	0.06	-1.69	1.12
Asn205	-2.37	-0.30	-2.33	-0.98	0.59	-0.04	-3.51	-0.63	-2.04	-0.60
Arg206	-0.20	0.06	-0.16	0.01	-0.16	0.00	-0.22	0.00	-0.13	-0.09
Val207	-0.12	-0.09	-0.15	-0.03	-0.14	-0.05	-0.16	-0.04	-0.12	0.00
His208	-2.68	0.32	0.48	0.94	-4.46	0.99	-4.37	0.21	-2.13	0.98
Arg209	-1.19	-0.44	-1.02	-0.29	-0.74	0.39	-1.45	-0.72	-1.10	-0.14
Trp210	-0.04	-0.07	-0.04	-0.02	-0.04	-0.02	-0.06	-0.07	-0.04	0.03
Val211	-0.04	-0.04	-0.05	-0.02	-0.04	-0.03	-0.05	0.04	-0.05	0.09
Gly212	-0.04	0.01	-0.04	0.07	-0.04	0.16	-0.04	0.13	-0.04	-0.07
Gly213	-0.03	-0.15	-0.03	-0.08	-0.03	-0.07	-0.03	-0.23	-0.03	-0.05
Gln214	-0.19	-0.50	-0.13	-0.01	-0.16	0.11	-0.14	-0.12	-0.13	-0.19
Met215	-0.87	-0.21	-1.08	-0.08	-1.06	0.25	-1.17	-0.41	-1.07	-0.80

Gly216	-0.87	-0.03	-1.46	-0.36	-1.22	-0.25	0.23	-4.99	-1.42	0.67
Val217	-0.22	-2.92	-1.84	0.58	-2.07	0.57	-1.94	0.67	-0.01	-0.52
Val218	-2.69	-0.13	-2.94	0.54	-3.50	0.65	-3.25	0.44	0.09	-0.73
Pro219	-0.24	0.14	-0.18	0.08	-0.23	0.05	-0.20	0.08	-0.24	-0.05
Thr220	-0.12	0.03	-0.10	0.02	-0.13	-0.11	-0.10	0.02	-0.11	-0.04
Ala221	0.43	0.20	-0.93	-0.45	0.00	-0.85	-0.69	-0.49	-0.90	0.40
Pro222	-0.14	-0.02	-0.13	-0.08	-0.17	-0.14	-0.16	-0.07	-0.18	0.07
Asn223	-0.02	0.10	-0.02	-0.09	-0.02	-0.20	-0.02	-0.06	-0.02	0.15
Asp224	-0.02	0.04	-0.02	0.05	-0.03	0.08	-0.02	0.02	-0.03	-0.02
Pro225	-0.01	0.03	-0.01	0.02	-0.01	0.03	-0.01	0.02	-0.01	0.01
Val226	-0.02	0.04	-0.02	0.00	-0.02	-0.01	-0.02	0.00	-0.02	0.03
Phe227	-0.49	-0.03	-0.72	-0.18	-0.77	-0.28	-0.73	-0.20	-0.93	0.29
Phe228	-0.02	0.07	-0.02	0.06	-0.02	0.09	-0.02	0.04	-0.03	0.03
Leu229	-0.01	0.00	-0.01	-0.02	-0.01	-0.03	-0.01	-0.01	-0.01	0.04
His230	-0.12	-0.25	-0.18	-0.17	-0.16	-0.21	-0.16	-0.07	-0.18	0.11
His231	-0.26	0.45	-0.37	0.58	-0.33	0.69	-0.31	0.21	-0.70	0.71
Ala232	0.00	0.00	0.00	-0.02	0.00	-0.03	0.00	-0.01	-0.01	0.03
Asn233	-0.01	-0.08	-0.01	-0.05	-0.01	-0.05	-0.01	-0.03	-0.01	0.02
Val234	-0.02	-0.06	-0.03	-0.05	-0.03	-0.07	-0.03	-0.01	-0.04	-0.02
Asp235	0.00	-0.02	0.00	-0.02	0.00	-0.04	0.00	-0.01	-0.01	0.00
Arg236	0.00	-0.05	0.00	-0.03	0.00	-0.04	0.00	-0.02	0.00	-0.01
Ile237	0.00	-0.03	0.00	-0.01	0.00	-0.01	0.00	-0.01	0.00	-0.01
Trp238	0.00	-0.02	0.00	-0.01	0.00	-0.03	0.00	0.00	0.00	-0.01
Ala239	0.00	-0.01	0.00	-0.01	0.00	-0.01	0.00	0.00	0.00	0.00
Val240	0.00	-0.01	0.00	0.00	0.00	0.00	0.00	0.00	0.00	0.00
Trp241	0.00	-0.01	0.00	0.00	0.00	0.00	0.00	0.00	0.00	-0.01
Gln242	0.00	0.01	0.00	0.01	0.00	0.01	0.00	0.01	0.00	-0.01
Ile243	0.00	-0.01	0.00	0.00	0.00	-0.01	0.00	0.00	0.00	0.00
Ile244	0.00	-0.01	0.00	0.00	0.00	0.00	0.00	0.00	0.00	0.00
His245	0.00	0.01	0.00	0.00	0.00	0.00	0.00	0.00	0.00	0.00
Arg246	0.00	0.00	0.00	0.00	0.00	-0.01	0.00	0.00	0.00	0.00
Asn247	0.00	0.00	0.00	0.00	0.00	0.00	0.00	0.00	0.00	0.00
Gln248	0.00	0.00	0.00	0.00	0.00	0.00	0.00	0.00	0.00	0.00
Asn249	0.00	0.00	0.00	-0.01	0.00	-0.01	0.00	0.00	0.00	0.01
Tyr250	0.00	-0.01	0.00	-0.01	0.00	-0.02	0.00	0.00	0.00	0.00
Gln251	0.00	-0.01	0.00	-0.01	0.00	0.00	0.00	-0.01	0.00	0.01
Pro252	0.00	0.00	0.00	0.00	0.00	0.01	0.00	0.00	0.00	0.00
Met253	0.00	0.00	0.00	0.00	0.00	0.01	0.00	0.00	0.00	0.01
Lys254	0.00	0.00	0.00	0.00	0.00	0.00	0.00	0.00	0.00	0.00
Asn255	0.00	0.00	0.00	0.00	0.00	-0.01	0.00	0.00	0.00	0.00
Gly256	0.00	0.01	0.00	0.02	0.00	0.01	0.00	0.01	0.00	-0.01
Pro257	0.00	0.01	0.00	0.00	0.00	0.01	0.00	0.00	0.00	0.00
Phe258	0.00	-0.01	0.00	-0.02	0.00	-0.01	0.00	-0.02	0.00	0.01
Gly259	0.00	0.00	0.00	-0.01	0.00	0.00	0.00	-0.01	0.00	0.01
Gln260	0.00	0.03	0.00	0.02	0.00	0.01	0.00	0.02	0.00	0.01
Asn261	0.00	0.00	0.00	-0.01	0.00	0.00	0.00	-0.01	0.00	0.01
Phe262	0.00	0.00	0.00	0.00	0.00	0.01	0.00	0.00	0.00	0.00
Arg263	0.00	0.00	0.00	0.01	0.00	0.01	0.00	0.00	0.00	0.00
Asp264	0.00	0.00	0.00	-0.01	0.00	0.00	0.00	0.00	0.00	0.01
Pro265	0.00	-0.01	0.00	-0.02	0.00	-0.01	0.00	-0.01	0.00	0.00
Met266	0.00	0.01	0.00	0.01	0.00	0.01	0.00	0.00	0.00	0.01
Tyr267	0.00	0.02	0.00	0.00	0.00	0.02	0.00	-0.01	0.00	0.02
Pro268	0.00	0.00	0.00	0.00	0.00	-0.01	0.00	0.01	0.00	-0.01
Trp269	0.00	-0.02	0.00	-0.02	0.00	-0.03	0.00	-0.01	-0.01	0.00
Asn270	0.00	-0.01	0.00	-0.01	0.00	-0.01	0.00	-0.01	0.00	0.01
Thr271	0.00	0.00	0.00	-0.01	0.00	0.00	0.00	-0.01	0.00	0.00
Thr272	0.00	0.01	0.00	0.01	0.00	0.02	0.00	0.01	0.00	0.00
Pro273	0.00	0.01	0.00	0.01	0.00	0.01	0.00	0.01	0.00	0.00

Glu274	0.00	0.01	0.00	0.01	0.00	0.01	0.00	0.00	0.00	0.00
Asp275	0.00	0.00	0.00	0.01	0.00	0.00	0.00	0.00	0.00	-0.01
Val276	0.00	0.01	0.00	0.00	0.00	0.00	0.00	0.00	0.00	0.00
Met277	0.00	0.01	0.00	0.00	0.00	0.00	0.00	0.00	0.00	0.01
Asn278	0.00	0.01	0.00	0.00	0.00	0.01	0.00	0.00	0.00	0.00
His279	0.00	0.00	0.00	0.01	0.00	0.01	0.00	0.00	0.00	-0.01
Arg280	0.00	0.00	0.00	0.00	0.00	0.00	0.00	0.00	0.00	0.00
Lys281	0.00	0.00	0.00	0.00	0.00	0.00	0.00	0.00	0.00	-0.01
Leu282	0.00	0.00	0.00	0.00	0.00	0.00	0.00	0.00	0.00	-0.01
Gly283	0.00	0.00	0.00	0.00	0.00	0.00	0.00	0.00	0.00	0.00
Tyr284	0.00	-0.01	0.00	-0.02	0.00	-0.02	0.00	-0.01	0.00	0.00
Val285	0.00	0.00	0.00	0.00	0.00	0.01	0.00	0.00	0.00	0.00
Tyr286	0.00	0.00	0.00	-0.01	0.00	-0.01	0.00	0.00	0.00	0.01
Asp287	0.00	-0.01	0.00	0.00	0.00	-0.01	0.00	0.00	0.00	0.00
Ile288	0.00	-0.01	0.00	0.00	0.00	-0.01	0.00	0.00	0.00	0.00
Glu289	0.00	-0.01	0.00	-0.01	0.00	-0.01	0.00	-0.01	0.00	0.00
Leu290	0.00	0.00	0.00	0.00	0.00	0.00	0.00	0.00	0.00	0.00
CuB291	-0.19	-2.26	-0.71	-1.42	-0.79	-7.77	-0.90	1.07	-3.52	-7.47
CuA292	-1.21	-9.37	-2.14	-8.59	-1.16	-9.91	-1.28	-2.50	-7.48	-5.22
